# Association mapping with a diverse population of *Puccinia graminis* f. sp. *tritici* identified avirulence loci interacting with the barley *Rpg1* stem rust resistance gene

**DOI:** 10.1186/s12864-024-10670-y

**Published:** 2024-08-01

**Authors:** Arjun Upadhaya, Sudha G. C. Upadhaya, Robert Brueggeman

**Affiliations:** Department of Crop and Soil Sciences, Washington State University, Pullman, WA 99164-6420 USA

**Keywords:** Wheat stem rust, Barley, *Puccinia graminis* f. sp. *tritici*, GWAS, Effector, *Rpg1*

## Abstract

**Background:**

Wheat stem rust, caused by *Puccinia graminis* f. sp. *tritici* (*Pgt*), is an important disease of barley and wheat. A diverse sexual *Pgt* population from the Pacific Northwest (PNW) region of the US contains a high proportion of individuals with virulence on the barley stem rust resistance (*R*) gene, *Rpg1*. However, the evolutionary mechanisms of this virulence on *Rpg1* are mysterious considering that *Rpg1* had not been deployed in the region and the gene had remained remarkably durable in the Midwestern US and prairie provinces of Canada.

**Methods and results:**

To identify *AvrRpg1* effectors, genome wide association studies (GWAS) were performed using 113 *Pgt* isolates collected from the PNW (*n* = 89 isolates) and Midwest (*n* = 24 isolates) regions of the US. Disease phenotype data were generated on two barley lines Morex and the Golden Promise transgenic (H228.2c) that carry the *Rpg1* gene. Genotype data was generated by whole genome sequencing (WGS) of 96 isolates (PNW = 89 isolates and Midwest = 7 isolates) and RNA sequencing (RNAseq) data from 17 Midwestern isolates. Utilizing ~1.2 million SNPs generated from WGS and phenotype data (*n* = 96 isolates) on the transgenic line H228.2c, 53 marker trait associations (MTAs) were identified. Utilizing ~140 K common SNPs generated from combined analysis of WGS and RNAseq data, two significant MTAs were identified using the cv Morex phenotyping data. The 55 MTAs defined two distinct avirulence loci, on supercontig 2.30 and supercontig 2.11 of the *Pgt* reference genome of *Pgt* isolate CRL 75-36-700-3. The major avirulence locus designated *AvrRpg1A* was identified with the GWAS using both barley lines and was delimited to a 35 kb interval on supercontig 2.30 containing four candidate genes (*PGTG_10878*, *PGTG_10884*, *PGTG_10885*, and *PGTG_10886*). The minor avirulence locus designated *AvrRpg1B* identified with cv Morex contained a single candidate gene (*PGTG_05433*). *AvrRpg1A* haplotype analysis provided strong evidence that a dominant avirulence gene underlies the locus.

**Conclusions:**

The association analysis identified strong candidate *AvrRpg1* genes. Further analysis to validate the *AvrRpg1* genes will fill knowledge gaps in our understanding of rust effector biology and the evolution and mechanism/s of *Pgt* virulence on *Rpg1*.

**Supplementary Information:**

The online version contains supplementary material available at 10.1186/s12864-024-10670-y.

## Background

*Puccinia graminis* f. sp. *tritici* (*Pgt*) is the causal agent of the disease wheat stem rust on barley (*Hordeum vulgare* L.) and wheat (*Triticum aestivum* L.) [1, 2]. Historically, *Pgt* caused economically devastating epidemics on both cereal crops around the globe [1, 3, 4]. During the second half of 20^th^ century, *Pgt* was managed through the deployment of effective genetic resistance in barley and wheat [4, 5]. The removal of the sexual alternate host, barberry (*Berberis vulgaris*), in the Midwestern United States (US) and Western Europe nearly eliminated the *Pgt* sexual cycle effectively stabilizing *Pgt* populations in these regions by limiting the development of new virulence gene recombination [1, 6]. However, the evolution of new virulent pathotypes like TTKSK (a.k.a Ug99) in Africa and the speed of dispersal across the continent reiterated the need for vigilant monitoring of *Pgt* sexual populations that can rapidly evolve virulence patterns that threaten global barley and wheat production [2, 7]. Recent stem rust outbreaks in Western Europe and their association with the sexual host, barberry, is alarming since genetic recombination during the sexual cycle can generate genotypes with novel virulence gene combinations [8–10].

The Pacific Northwest (PNW: US states Washington, Idaho, Oregon, and Canadian province British Columbia) region of North America serves as a center of *Pgt* diversity on the continent [11–15]. In the PNW, *Pgt* cycles between cereals, native grasses, Mahonia, and barberry shrubs to complete its sexual spore forming stage [12–15]. Recently, the native shrubs endemic to the woodland areas of the PNW, *Mahonia repens* and *Mahonia auilifolium*, were shown to serve as sexual hosts of *Pgt* in addition to *B. vulgaris* that survived eradication in the PNW [13]. Virulence characterization of *Pgt* isolates collected from Eastern Washington on the two major barley *R*-genes, *Rpg1* and the *rpg4/5-*mediated resistance locus (RMRL) [15] identified isolates that are virulent on barley line Q21861, which carries both *R*-genes. This was the first report of *Pgt* races or isolates that were virulent on both major barley stem rust *R*-genes when stacked together representing the most virulent *Pgt* population on barley from across the globe.

We hypothesized that genetic reshuffling during sexual reproduction of *Pgt* allowed both virulence genes to recombine in the PNW *Pgt* genotypes as isolates virulent on each *R*-gene are also present in the population at higher frequency than the isolates virulent on both *R*-genes. These virulent genotypes not only pose a threat to the Washington barley industry but could be wind disseminated over the Rocky Mountain and threaten production in other major barley producing regions of North America. Indeed, the *Pgt* race QCCJB, the first North American isolate with virulence on barley lines containing *Rpg1*, that caused epidemics on barley and wheat during 1990s in the Midwestern US was hypothesized to have originated from the PNW region [12].

*Rpg1*, RMRL, and the recently identified *Rpg7* are the three major wheat stem rust resistance genes in barley that confer all stage resistance to many North American pathotypes [5, 16–18]. Of these *R*-genes, *Rpg1* is the only one deployed in commercial barley cultivars grown in the Northern Great Plains and Canadian Prairie provinces [5, 16]. Since the 1950s *Rpg1* provided remarkably durable resistance for nearly 50 years, until *Pgt* race QCCJB emerged in the Northern Great Plains with virulence on barley lines containing *Rpg1* [5, 12]. *Rpg1* was mapped to the telomeric region of chromosome 7H [19] and cloned via a map-based strategy [16]. *Rpg1* encodes a dual kinase domain protein with a pseudokinase domain (pK1) and an active kinase domain (pK2), that are both required for resistance [16]. Previously, two effectors, RGD and VPS9 corresponding to the barley *Rpg1* gene were identified from *Pgt* race MCCF [20, 21]. Inoculation with *Pgt* race MCCF containing these effectors resulted in phosphorylation of RPG1 as early as 5 min post inoculation, before spore germination (30 min), suggesting that these effectors are very early elicitors of the *Rpg1*-mediated resistance response.

Stem rust virulence/avirulence effector identification has been slow partly due to the inability to culture the obligate biotrophic pathogen outside its hosts [22]. Till now, seven *Pgt* effector genes have been identified including *AvrSr35* [23], *AvrSr50* [24], *AvrSr27* [25], *AvrSr13c* [26], *AvrSr22* [26], *RGD* [20, 21], and *VPS9* [20, 21]. Several approaches including transcriptomics, bi-parental mapping, and comparative genomics have been used to identify candidate effector loci or genes in fungal pathogens [27–30]. However, advances in high-throughput DNA sequencing have enabled whole genome sequencing of fungal pathogens at the population level, producing high density SNP markers which allow for high-resolution mapping via genome wide association studies (GWAS). This advancement in the high-resolution genetic characterization of fungal plant pathogen populations for host-pathogen genetic interactions allows for the rapid identification of candidate virulence/avirulence effector genes [31–35]. GWAS has been extensively used in plants and animals [36] but recently became a powerful tool to genetically map virulence/avirulence loci in plant fungal pathogens [31–35, 37, 38]. For example, [32] conducted a GWAS study on a natural population of *Parastagonospora nodorum*, a necrotrophic fungal pathogen of wheat, and identified novel virulence loci along with the previously described effector genes, *SnToxA* and *SnTox3*. Similarly, in another haploid fungal pathogen of wheat, *Zymoseptoria tritici*, the avirulence gene *Zt_8_609* was discovered through genome wide association analyses [31]. Although the GWAS approach has increasingly been utilized in haploid plant fungal pathogen populations the approach has seen little utilization in the dikaryotic cereal rusts. Recently, a similar approach, transcriptome wide association study (TWAS), was performed using 24 *Pgt* isolates to identify 33 variants within 28 genes that were associated with virulence on the barley *rpg4/Rpg5*-mediated resistance locus (RMRL) [30]. In a separate GWAS using 96 *Pgt* isolates, 17 *Pgt* loci were found to be associated with six stem rust resistance genes in wheat [34, 35]. Once identified, avirulence or virulence effectors can be utilized to aid in the deployment of broad-spectrum resistances in crops by accelerating *R*-gene identification, identifying specific interactions with host virulence targets, and providing an effective means to screen breeding materials if an effective delivery method is available by delimiting single gene-for-gene interactions [39]. Effector characterization can also help elucidate evolutionary mechanism of virulence acquisition in pathogens [23–25, 40]. For example, allele analysis of *AvrSr35* among virulent and avirulent *Pgt* isolates identified that the insertion of a mobile element into *AvrSr35* resulted in virulence on the wheat *Sr35* gene [23].

The *Pgt* population characterized from eastern Washington in the PNW is dominated by genotypes that are virulent on barley lines containing *Rpg1* (99%), contain a high proportion of individuals virulent on RMRL (16%), and 10% of the population is virulent on barley containing both *Rpg1* and RMRL [15]. This raises the question of how this high level of virulence on barley became predominant in the population considering that neither the *Rpg1* nor RMRL resistances were deployed in the region. Thus, the overarching goal of this study was to identify candidate *Rpg1* virulence/avirulence effector genes in order to begin filling knowledge gaps in stem rust effector biology and the evolutionary processes that led to this high level of virulence on barley. Utilizing whole genome sequencing, RNAseq data and infection type data for 113 *Pgt* isolates, 55 significant MTAs were identified corresponding to two unique loci in the *Pgt* genome that putatively contain AvrRpg1 effectors. Thus, *Pgt* loci that evolved to overcome *Rpg1* resistance were characterized using genome wide association studies (GWAS) and candidate *AvrRpg1* genes underlying these loci identified.

## Methods

### Barley genotypes

Five barley genotypes including the transgenic line H228.2c and cultivars (cvs) Morex, Golden Promise, Harrington, and Steptoe were used in this study (Table 1). The Golden Promise transgenic line (H228.2c) and cv Morex (CIho 15773) represent genotypes containing *Rpg1* in distinct genetic backgrounds. Morex is a natural source of the *Rpg1* gene from which the gene was originally cloned [16]. The Golden Promise transgenic line (H228.2c) carries a single copy *of the* *Rpg1* gene from cv Morex in the susceptible cv Golden Promise (GP) background [41] (Table 1). Wildtype cv Golden Promise (PI 343079) is a two-rowed malting barley developed by the Miln Marsters seed company Cheshire, UK using mutational breeding. Morex, is a six-rowed malting variety released by the University of Minnesota [42]. Steptoe (CIho 15229) is a six-rowed feed barley released by Washington State University [43]. Harrington is a two-rowed spring malting barley developed at the University of Saskatchewan [44]. Barley cultivars Steptoe, Harrington, and Golden Promise were used as susceptible checks because they are considered universal stem rust susceptible checks that do not carry any known stem rust *R*-genes [15] (Table 1). Details on the source, improvement status, and *R*-gene of the plant materials are provided in Table 1.

**Table 1:** Source, improvement status, and stem rust *R*-gene present in the barley genotypes used in this study

**S.N**	**Plant material**	**Developer or source**	**Improvement Status**	**R-gene**
1	Steptoe (CIho 15229)	Washington State University, USA	Cultivar	None
2	Harrington	University of Saskatchewan, Canada	Cultivar	None
3	Golden Promise (GP) (PI 343079)	Miln Marsters seed company, Cheshire, UK	Cultivar	None
4	GP transgenic line (H228.2c)	Washington State University, USA [41]	Transgenic (*Rpg1*)	*Rpg1* +
5	Morex (CIho 15773)	University of Minnesota, USA	Cultivar	*Rpg1* +

### *Puccinia graminis* f. sp. *tritici* isolates

A total of 113 *Pgt* isolates were utilized in this study, including 89 from the Pacific Northwest region (PNW) (Washington and Idaho) and 24 from the Midwest (North Dakota) region of the U.S (Supplementary Table S1). The PNW isolates were derived from stem rust samples collected from diverse hosts including barley, wheat, *Mahonia*, and barberry during the 2019–2020 growing seasons (Supplementary Table S1). The Midwestern isolates were collected as part of cereal rust surveys conducted in North Dakota from 1977 to 1999. Details on isolates are presented in Supplementary Table S1.

### Phenotype assays

Disease phenotype data for 96 of the *Pgt* isolates was generated based on their infection types on the barley lines; WT cv Golden Promise (*Rpg1* -), the cv Golden Promise transgenic line H228.2c (*Rpg1* +), cv Morex (*Rpg1* +), cv Steptoe (*Rpg1* -), and cv Harrington (*Rpg1* -). We also utilized previously generated phenotype data on cv Morex for 17 additional Midwest isolates. Thus, disease phenotypes on cv Morex (*Rpg1* +) was obtained for 113 *Pgt* isolates. Two *Pgt* races QCCJB and HKHJ with known disease reactions on the aforementioned barley lines were used as controls. *Pgt* race QCCJB is virulent and *Pgt* race HKHJ is avirulent on barley lines containing *Rpg1* [15].

Two seeds of each barley line were grown in containers filled with potting soil mix (Sungro Horticulture, MA, USA). Host genotypes were replicated three times in a completely randomized design. Each phenotype assay was conducted twice. The seedlings were grown in environmentally controlled growth chambers set at 19 ± 1ºC with a 16-h (400 µm/m^2^) light and 8 h dark cycle. Inoculations were performed when primary leaves were fully expanded (~ 9 days after planting). Seedlings were inoculated with a suspension of light mineral oil and urediniospores (0.05 mg urediniospore/plant) using atomizers pressured by a pump set at 30 kPa [15]. After inoculation, plants were kept in mist chambers set at 19ºC, and 100% RH with complete darkness for 18 h to facilitate the infection process. Seedlings were returned to the growth chamber and disease ratings taken at 14 days after inoculations (DAI).

Stem rust infection types (ITs) were assessed for primary leaves at 14 DAI using the “modified 0–4 scale”, originally developed for wheat by [45]. The modification of this 0–4 scale for barley were described by [46] and were based on the uredinial sizes on barley leaves. The description of each IT on the 0–4 scale for barley is explained by [47]. Barley shows a mesothetic reaction to *Pgt* where multiple ITs are observed on a single leaf. In this case, ITs were recorded in their order of prevalence. Then, the categorical phenotype scores of 0 to 4 were converted into numeric quantitative scores of 0 to 5 for the ease of virulence interpretation and association analysis [48]. A numeric quantitative disease score of < 3 was considered avirulent, and > 3 as virulent.

### *Puccinia graminis* f. sp. *tritici* genotyping

#### Pathogen DNA extraction

Genomic DNA (gDNA) of each isolate (*n* = 96) was extracted from urediniospores (~ 30 mg/isolate) using the Quick-DNA Fungal/Bacterial Miniprep kit (Zymo Research) following the manufacturer’s recommended protocol. The integrity of gDNA was assessed on 1.2% agarose gel stained with gel red. gDNA with a high molecular weight band (~ 10–15 kb) without smearing was considered as high-quality gDNA. gDNA for each isolate was quantified on the Qubit 4.0 fluorometer using the Broad Range assay kit (ThermoFisher Scientific). The purity of gDNA samples were assessed using a NanoDrop 1000 Spectrophotometer (ThermoFisher Scientific). gDNA samples with 260/280 ratio of 1.8 ± 0.1 and 260/230 ratio of 2 ± 0.1 or above were considered pure DNA samples and utilized for whole genome sequencing library preparation.

#### WGS library preparation and sequencing

Whole genome shotgun sequencing libraries were prepared for each of 96 isolates using the FS DNA library prep kit (NEB, New England Biolabs) following the manufacturer’s recommendations (Table 2). Briefly, gDNA (500 ng/sample) was randomly sheared, adaptor-ligated, size-selected, barcoded, PCR enriched, and bead cleaned to generate libraries with 250–300 bp insert sizes. Each isolate library was barcoded with unique dual indexes to facilitate multiplexing of samples for sequencing. Library fragment sizes were determined using the DNA 1000 assay on an Agilent 2100 bioanalyzer system (Agilent technologies). Libraries with a single main peak around 370–420 bp and without primer (80 bp) and adapter-dimer (128 bp) peaks were considered quality libraries for sequencing. Libraries (*n* = 96) were normalized to a 5 nM concentration which was based on the average fragment size (bp) as determined by bioanalyzer data and DNA concentrations measured by the Qubit broad assay. Normalized libraries were pooled together and an aliquot of 50 µl at 5 nM concentration was sent to Novogene corporation (Sacramento, CA, USA) for sequencing. At Novogene, the concentration of effective library was determined by qPCR assays before sequencing. A 150 bp paired-end sequencing run was performed utilizing a single lane on a Novoseq 6000 sequencer (Illumina platform).

**Table 2:** Summary of sequencing strategy for *Puccinia graminis* f. sp. *tritici* isolates used in this study^a^

**Number of isolates**	**Genotyping**	**Location**
89	WGSS	WA & ID
7	WGSS	ND
**96 (sub-total)**		
17	RNA-seq	ND
**113 (Total)**		

^a^A total of 96 isolates were genotyped by whole-genome shotgun sequencing (WGSS) approach. Seventeen isolates from ND, Midwest were genotyped by RNA sequencing approach [30]. WA, ID, and ND refer to the states of Washington, Idaho, and North Dakota, respectively, from where isolates were collected

#### Quality control, mapping and variant calling for WGS data

The quality of raw sequencing reads was examined using FastQC (v0.11.9) and low-quality reads were filtered out using Fastp software (v0.22.0) [49]. The *Pgt* isolate CRL 75-36-700-3 reference genome was indexed using Burrows-Wheeler Alignment (BWA) tool (v0.7.17). The *Pgt* isolate CRL 75-36-700-3 genome assembly represents the collapsed haploid assembly of two karyons of dikaryotic urediniospores with 392 supercontigs and a total assembly size of 88.72 Mbp [50]. Quality reads were mapped to the indexed reference genome using the BWA-mem algorithm with default settings [51]. Alignment files in BAM format were coordinate sorted, indexed, and then duplicates were marked and removed using the Picard tools (v2.25.4). Variants were detected using the GATK (v4.2.5.0) tool. Briefly, per sample variant calling was done using GATK HaplotypeCaller then calls for all samples were merged using CombineGVCFs, and finally joint variants were called with the GenotypeGVCFs function of GATK. The raw variants were split into SNPs and INDELs using the SelectVariants function of GATK. Variants were filtered in two steps, first using the GATK hard filtering function and second using the VCFtools to generate a suite of high-quality variants. Hard filtering was done using GATK VariantFiltration with options “QD < 2.0 || MQ < 40.0 || FS > 60.0 || SOR > 4.0 || MQRankSum < -12.5 || ReadPosRankSum < -8.0” for SNP filtering and "QD < 2.0 || FS > 200.0 || ReadPosRanksum < -20" for INDEL filtering. The SNPs and INDELs that passed the above criteria were filtered again using the VCFtools with parameter settings as: --minQ 30 --maf 0.05 --min-alleles 2 --max-alleles 2 --max-missing 1 --minDP 3 --min-meanDP 5. This generated a collection of high-quality variants for the 96 *Pgt* isolates sequenced using the WGS approach. SNP variants were utilized for associating mapping with GPT, population structure and relatedness assessment, and linkage disequilibrium calculation.

#### RNAseq library preparation and sequencing

RNA extraction, cDNA synthesis, library preparation and sequencing for 17 Midwest isolates was previously reported [30] (Table 2). In this study we utilized previously generated transcript sequence data to identify variants for 17 Midwest *Pgt* isolates.

#### Quality control, mapping and variant calling for RNAseq data

Quality control of raw sequencing reads was done as described for the WGS data. Quality reads were mapped to the CRL 75-36-700-3 reference genome using STAR mapper (v2.7.10a), a splice aware alignment tool [52]. Briefly, a genome index was build utilizing both reference assembly and gene annotation. Then, trimmed reads were mapped to the reference genome with option “--twopassMode Basic”, to improve alignment to the splice junctions. Alignment files in BAM format were coordinate sorted and indexed as described for the WGS data. Variant calling was done using GATK (v4.2.5.0). Before variant calling, RNAseq alignment was reformatted using the SplitNCigarReads function of GATK. This generated an alignment where reads containing Ns at the splicing events were split and mapping qualities reassigned to match DNA convention for GATK, HaplotypeCaller. Per sample variants were called using Haplotypecaller. Then, the sample variants of the 17 Midwestern isolates were merged with variants of 96 whole genome sequenced PNW isolates. Finally, joint variant calling was done for all 113 isolates using the GenotypeGVCFs function of GATK. Then, raw variants were processed further following the same procedure and parameters described for WGS. SNP variants obtained by combining RNAseq and WGS data were used for identification of marker-trait associations (MTAs) with IT data generated for cv Morex.

### Variants effect prediction

The SNP and Indel effects were determined and annotated using SnpEff (v5.1) [53]. The SnpEff tool categorizes variant effects by impact into four classes: High, Moderate, Low, and Modifier. The High and Moderate impact variants were considered detrimental, and their densities were determined on each supercontig (*n* = 392) of the CRL 75-36-700-3 reference genome. A detailed explanation on different types of variants under these four classes can be found at http://snpeff.sourceforge.net/VCFannotationformat_v1.0.pdf.

### Variant heterozygosity and homozygosity

The proportion of homozygous and heterozygous SNP variants for the 96 *Pgt* isolates was determined using “stats” function of bcftools (v1.10.2) [54].

### Principal component analysis (PCA)

PCA was performed on a reduced set of SNPs because of the computational load and run times with large data sets. Ten percent of the SNPs were randomly selected using the SelectVariants function of GATK. PCA was performed using the package SNPRelate [55] to describe the population structure of *Pgt* isolates (*n* = 96) for which genome wide SNP variants were available. The percentage of variance explained by each principal component was determined using the eigen values from PCA result. Finally, new projected dimensions were visualized using the ggplot2 package in R.

### *Pgt* isolates relatedness

Relatedness among the *Pgt* isolates was assessed utilizing 10% randomly distributed SNPs because of computational difficulty to handle a large distance matrix. Genetic distance between *Pgt* isolates (*n* = 96) was calculated using the ‘gdist’ function of the NAM package [56] in R. Based on the genetic distances of *Pgt* isolates clustering was performed using the hclust function and ward method in R. A dendrogram was constructed using “dendextend” package [57] with minimum number of clusters (k = 3) based on the lower Bayesian Information Criterion (BIC) value to evaluate and visualize relatedness among isolates. Finally, a dendrogram was generated in circular form using the circlize package [58] in R.

### Linkage disequilibrium (LD) calculation

LD was computed as squared allele frequency correlations (*R*^2^) between intrasupercontig marker pairs using a sliding window size of 50 markers surrounding the current site in the Tassel software (v5.0) [59]. LD was performed on a reduced marker set (10%) for computational efficiency. To understand the pattern of LD decay, a non-linear model, y = log(x) was fitted, where x denotes distance between marker pairs (in kb) and y denotes *R*^2^ value between marker pairs [34, 35].

### Genome wide association studies (GWAS)

Association analysis between genotype and phenotype was performed using genome association and prediction integrated tool (GAPIT) (v3.3) [60]. Quantitative disease scores were utilized as phenotype data and SNPs as genotype data. The mixed linear model (MLM), which can incorporate principal components (PCs), (Q) as fixed effect covariate and kinship/relatedness (K) as random effect covariate was run for association analysis [61]. The number of PCs that explained at least 25% variation were included in the model. Similarly, GAPIT by default incorporated the kinship data generated with the VanRaden function within the MLM model. If no significant association was detected with the MLM model, an additional model, BLINK was run. For the BLINK model [62], only the PCs could be incorporated as covariates. The Bonferroni correction was applied to *p*-value (0.05) to prevent false association due to multiple testing of markers. The corrected *p*-value was calculated by dividing the generic *p*-value (0.05) by the total number of tested markers. The markers were considered significantly associated with phenotype only when the *p*-value associated with markers was lower than the corrected *p*-value.

### Candidate effector identification

The logarithm of the odd (LOD) scores were calculated [LOD = -log10(*p*-value)] for all significant MTA SNP markers present on the *Pgt* isolate CRL 75-36-700-3 reference genome supercontigs. The LOD scores were plotted along the physical position of markers to identify regions that harbor significant MTA (Supplementary Fig. S1). SNP markers flanking the significant MTA that fell below the significant threshold level were identified as flanking markers to delimit the physical regions containing candidate *AvrRpg1* genes (Supplementary Fig. S1). The predicted gene models within the delimited regions were identified based on the *Pgt* isolate CRL 75-36-700-3 reference genome [50] annotations and designated as candidate *AvrRpg1* genes.

### Characterization of candidate loci, underlying genes, and alleles

SNP and Indel densities for candidate *AvrRpg1* loci were calculated to determine variant coverage in the region. Repeat features in the delimited *AvrRpg1* region/loci were extracted using Ensembl REST API to visualize repeat landscape. Because the *Pgt* isolate CRL 75-36-700-3 reference genome supercontigs are not anchored at the chromosome level, candidate *AvrRpg1* gene sequences were BLAST (Basic Local Alignment Search Tool) searched against the genome assembly of the Australian *Pgt* isolate Pgt21-0 [40] for which a haplotype phased chromosome level assembly is available. Briefly, the Pgt21-0 genome assembly was obtained from the JGI MycoCosm web portal (https://mycocosm.jgi.doe.gov/mycocosm/home/releases?flt=puccinia+graminis) and a local nucleotide blast database was created using the makeblast function of the BLAST (v2.5.0 +) toolkit. Candidate gene sequences were blasted against the local Pgt21-0 nucleotide database using the blastn function of BLAST to determine presence/absence and chromosomal location of genes. Protein homologs of candidate genes were searched using the BLASTP program in the NCBI protein database at a threshold level of 90% identify with 90% query coverage. We also blast searched the candidate gene sequences against the reference assembly to find if these genes evolved through duplication events. Significant MTAs were aligned and manually assessed to determine the haplotype state, homozygous vs heterozygous reference or alternate allele, for each locus among virulent and avirulent isolates to predict the dominant nature of avirulence or virulence. For the most significant SNP on each gene, the correlation between SNP haplotypes and phenotypes was computed using the point bi-serial method in R. A Kruskal-Wallis test was performed to determine if the haplotypes of the most significant SNP on each gene differed statistically for disease phenotypes.

## Results

### Pathogen phenotype

**Fig. 1:**
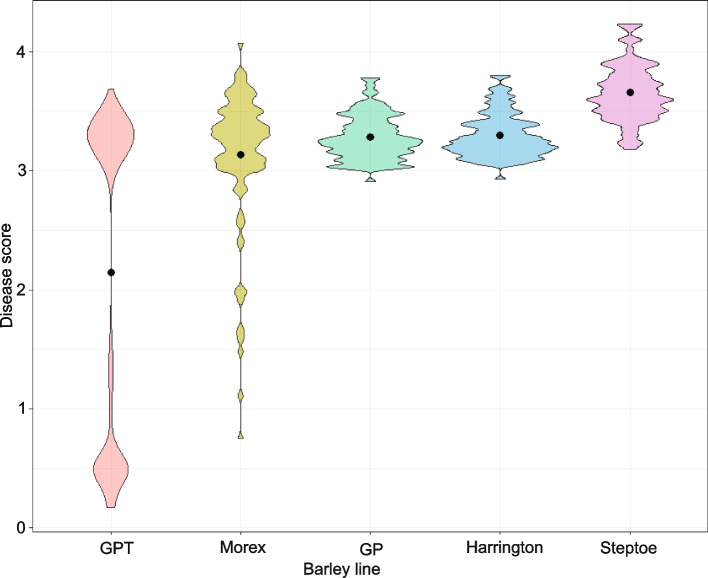
Disease phenotype distribution of the *Pgt* isolates used in this study. Black dot in each violin indicates mean disease score. Disease scores are presented on a 0–5 scale. *The Pgt* population showed a clear unimodal disease distribution on susceptible lines, Steptoe, Harrington and Golden Promise (GP). A bimodal disease distribution was observed on Golden promise transgenic line (H228.2c), carrying the cv Morex source of the *Rpg1* gene. A skewed distribution was observed on Morex, carrying the natural source of the *Rpg1* gene. Barley lines, Golden Promise and Golden Promise Transgenic are abbreviated as GP and GPT, respectively in the violin plot

Each isolate was phenotyped for disease infection types (ITs) on the five barley genotypes; WT cv Golden Promise (*Rpg1* -), the cv Golden Promise transgenic line H228.2c (*Rpg1* +), cv Morex (*Rpg1* +), cv Steptoe (*Rpg1* -), and cv Harrington (*Rpg1* -). For the ease of virulence interpretation, ITs were converted to numeric disease scores of 0–5. Fifty-seven percent of the isolates (*n* = 96) were virulent on the cv Golden Promise transgenic line H228.2c (*Rpg1* +) (Fig. 1, Supplementary Table S2). Disease scores of the 96 *Pgt* isolates on H228.2c ranged from 0.17 to 3.69, with an average of 2.14. (Fig. 1). Eighty-three percent of the isolates (*n* = 113) showed virulence on cv Morex (*Rpg1* +), carrying the natural source of the *Rpg1* gene (Fig. 1, Supplementary Table S2). Disease scores of the 113 *Pgt* isolates on cv Morex ranged from 0.75 to 4.07, with a mean of 3.14 (Fig. 1). H228.2c exhibited a strong immune reaction (ITs- 0, 0; or 0;1) against all avirulent isolates while cv Morex did not show these high levels of resistance in response to the avirulent isolates (Fig. 1). Two control *Pgt* races QCCJB and HKHJ exhibited disease phenotype as expected where *Pgt* race QCCJB was virulent on H228.2c and cv Morex and *Pgt* race HKHJ was avirulent on both H228.2c and cv Morex. The susceptible checks, cvs Steptoe, Harrington, and Golden Promise, displayed susceptible reactions to *Pgt* races QCCJB and HKHJ as expected. However, the average disease score of the *Pgt* population was comparatively higher on Steptoe than Harrington and Golden Promise (Fig. 1).

### Sequencing and mapping statistics

**Fig. 2:**
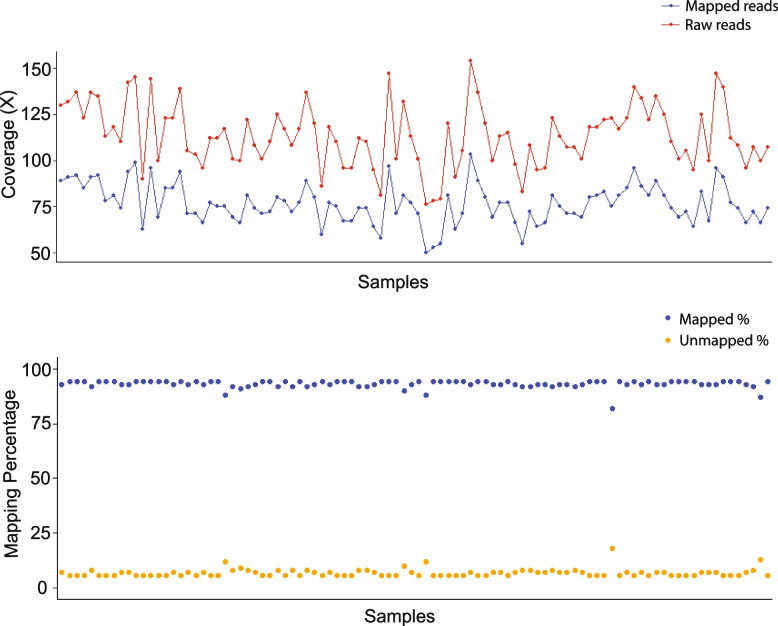
Read coverage and mapping summary for *Pgt* isolates (*n* = 96) sequenced using the whole genome sequencing approach. **A** Red line with dots indicates the genome coverage computed for each *Pgt* isolate using raw sequencing reads. Blue line with dots shows the genome coverage computed with mapped reads after quality control. **B** Blue and yellow dots indicate the percentage of mapped and unmapped reads, respectively to the reference genome, CRL 75-36-700-3. Each dot represents a *Pgt* isolate

Whole genome sequencing of the 96 *Pgt* isolates primarily collected from the PNW yielded a total of 6.4 billion paired end (PE) reads on a single Novogene 6000 (Illumina) lane. The number of raw reads ranged from 45 to 91 million per sample with an average of 67 million. This led to an average estimated genome coverage of 113 × of the *Pgt* genome as calculated by the *Pgt* isolate CRL 75-36-700-3 reference genome assembly size of 88.72 Mb (Fig. 2A). Raw reads were quality trimmed and mapped to the CRL 75-36-700-3 reference genome. The mapping rate ranged from 82 to 94% with an average of 93% (Fig. 2B). The estimated genome coverage when computed with mapped reads, ranged from 50 × to 106 × with an average of 79 × per sample (Fig. 2A). We extracted the unmapped reads from samples with < 90% mapping rate (*n* = 4). These reads predominantly represented thrip (*Frankliniella occidentalis*), barley (*Hordeum vulgare* Subsp. *vulgare*), and bacterial (*Pantoea* spp.) genomic DNA sequences. These contaminants were probably introduced during rust isolate increase and subsequent handling in the greenhouse.

For the 17 Midwest isolates, an RNAseq approach was used generating a total of 0.7 billion single end (SE) reads on an Illumina NextSeq 500 sequencer. The number of raw reads ranged from 33 to 82 million per sample with an average of 44 million. Seedling primary leaf tissues infected with each of the 17 Midwest *Pgt* isolates were utilized for RNA extraction, hence the raw data included both *Pgt* and barley RNAseq reads. The percentage of quality trimmed reads that mapped to the CRL 75-36-700-3 reference genome ranged from 5 to 69% per sample with an average of 43%.

### Variant statistics

**Fig. 3:**
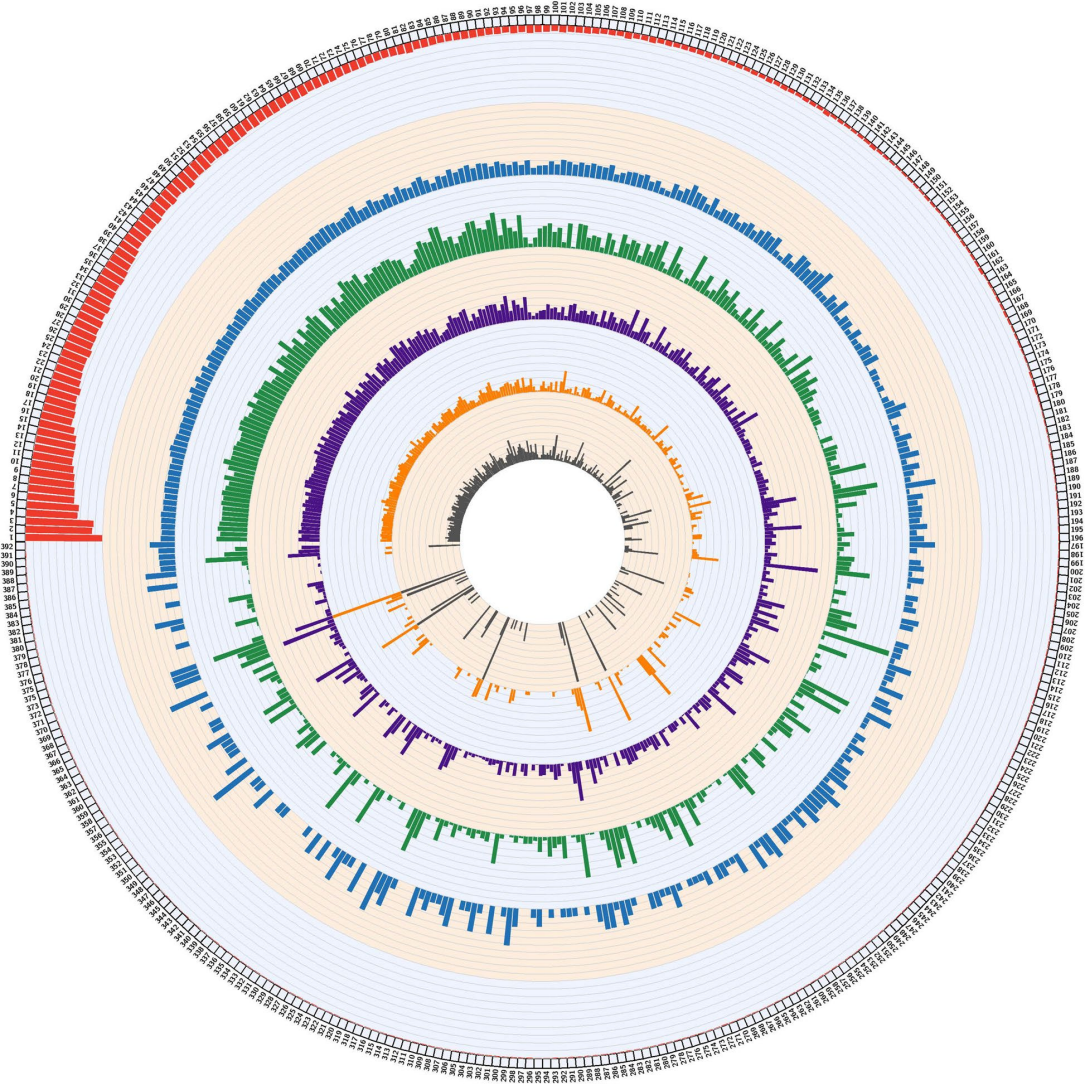
Genomewide distribution of variants (SNPs/Indels) identified among *Pgt* isolates (*n* = 96). Red bars on the outer layer indicate the length of supercontigs (*n* = 392 supercontigs) for the *Pgt* reference assembly, CRL-75-36-700-3. Each axis on this layer represents 300 kb. Blue bars show gene densities on each supercontig. Each axis represents 1 gene/10 kb. Green and purple bars indicate SNP and Indel densities, respectively. Each axis represents 4 SNPs or 1 Indel per kb. Orange bars display the densities of deleterious SNPs. Each axis represents 1 SNP per kb. Grey bars indicate the densities of deleterious Indels. Each axis represents 1 Indel per 10 kb. Deleterious refers to high and moderate impact variants based on the SnpEff tool

Mapping of WGS reads to the CRL-75-36-700-3 reference genome and subsequent variant calling identified 1,195,947 SNPs and 168,516 Indels among the 96 *Pgt* isolates. The densities of variants were computed on each supercontig (*n* = 392) to determine genome-wide variant distribution (Fig. 3). SNP densities varied from 0 to 38 SNPs/Kb across the supercontigs (Fig. 3). Smaller supercontigs (< 250 Kb) had uneven SNP and Indel densities compared to longer supercontigs (Fig. 3). The densities of Indels ranged from 0 to 8 Indels/Kb across the supercontigs (Fig. 3). The size of Indels extended from 1 to 60 bp in length with the most frequent being 1 bp (33%) followed by 2 bp (20%), and 3 bp (14%). Smaller Indels (< 10 bp) accounted for 91% of total identified Indels.

Joint variant calling using the WGS for the 96 *Pgt* isolates and RNAseq data for the 17 Midwestern isolates yielded a total of 136,391 SNPs and 8,238 Indels among the total of 113 *Pgt* isolates. Lower number of variants were detected in this combined analysis compared to only WGS data because the RNAseq alignments of the additional 17 isolates limited the variant detection to genic regions. Also, we did not permit any missing data, so variants identified outside genic regions based on WGS data were filtered out.

### Variants effect predictions

The effects of variants were predicted using SnpEff. This tool outputs all possible consequences that can be caused by a variant in a gene’s transcript isoforms or in multiple genes sharing the same promoter sequence. Thus, the total number of effects was greater than the total number of variants. The 1,195,947 SNPs and 168,516 Indels caused 4,012,622 and 643,357 effects, respectively. Variant effects were further categorized as high (0.07%), moderate (2.91%), low (4.60%), and modifier (92.4%) based on the impact they were predicted to cause in the genic regions. The densities of high and moderate impact SNPs were computed and visualized across each supercontig (Fig. 3). The ratio of missense to silent mutations was 0.71 for SNP variants. A total of 1,911 nonsense effects (stop gained) were caused by SNPs across the *Pgt* genome. The effects of SNPs were mostly in downstream (34%) and upstream regions (34%) followed by intergenic (18%), exon (7%), intron (3%), and others (4%). High, moderate, low, and modifier Indel effects accounted for 1.36%, 0.86%, 0.47%, and 97.29%, respectively, of the total predicted effects. Densities of high and moderate impact indels were calculated and visualized across each supercontig (Fig. 3). Indel effects were primarily in upstream (36%) and downstream (35%) regions followed by intergenic (19%), intron (4%), exon (2%) and others (4%). Indels caused a total of 8,250 frameshift mutation within predicted genes across the genome.

### Heterozygosity and homozygosity of SNP variants

**Fig. 4:**
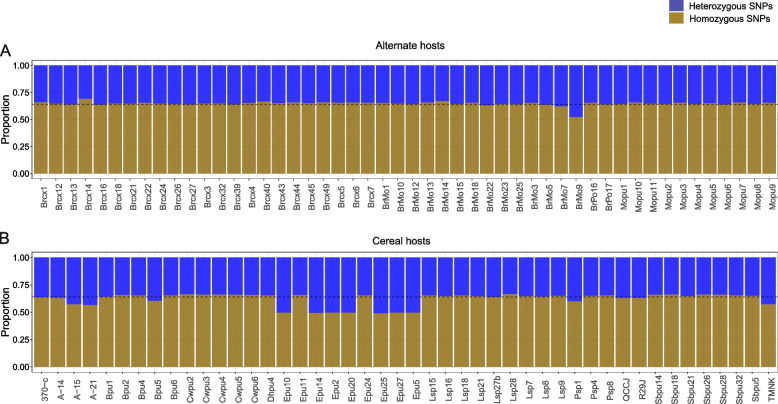
Bar charts depicting the proportion of homozygous and heterozygous SNP variants for *Pgt* isolates derived from **A** alternate hosts (*n* = 50) and **B** cereal hosts (*n* = 46). The gold and blue portion of each bar represents the proportion of homozygous and heterozygous SNP variants, respectively. Dotted lines indicate average homozygosity (0.63) for the *Pgt* population

To understand the genetic composition of *Pgt* isolates (*n* = 96), the proportions of homozygous and heterozygous SNP variants were determined. The rate of SNP homozygosity extended from 49 to 69% per isolate with an average of 63% for the population (Fig. 4). SNP heterozygosity ranged from 31 to 51% per isolate with a population average of 37% (Fig. 4). Among the isolates (*n* = 50) collected from the alternate hosts barberry and Mahonia, the average rate of homozygous and heterozygous SNP variants was 65% (52 to 69%) and 35% (31 to 48%) (Fig. 4). Among isolates collected from the cereal hosts, barley, and wheat (*n* = 46), the average proportion of homozygous and heterozygous variants was 62% (49 to 66%) and 38% (34 to 54%), respectively (Fig. 4). The six Midwest isolates (TMNK, 370-c, A-14, A-15, A-21, R29J) among the cereal isolates (*n* = 46) are believed to be asexual isolates [30]. The average rate of homozygous and heterozygous SNP variants was 60% (56 to 63%) and 40% (37 to 44%), respectively for the six Midwest *Pgt* isolates.

### Principal component analysis

Principal component analysis was done to infer the structure of the *Pgt* population (*n* = 96). The first three principal components (PCs) explained 26.5% of the variance in the *Pgt* population structure (Supplementary Fig. S2). The percentage of variance explained by PC1, PC2, and PC3 was 15.60%, 6.35%, and 4.55%, respectively. PC1 vs PC2 and PC2 vs PC3 were visualized with biplots. (Supplementary Fig. S2).

### Relatedness

**Fig. 5:**
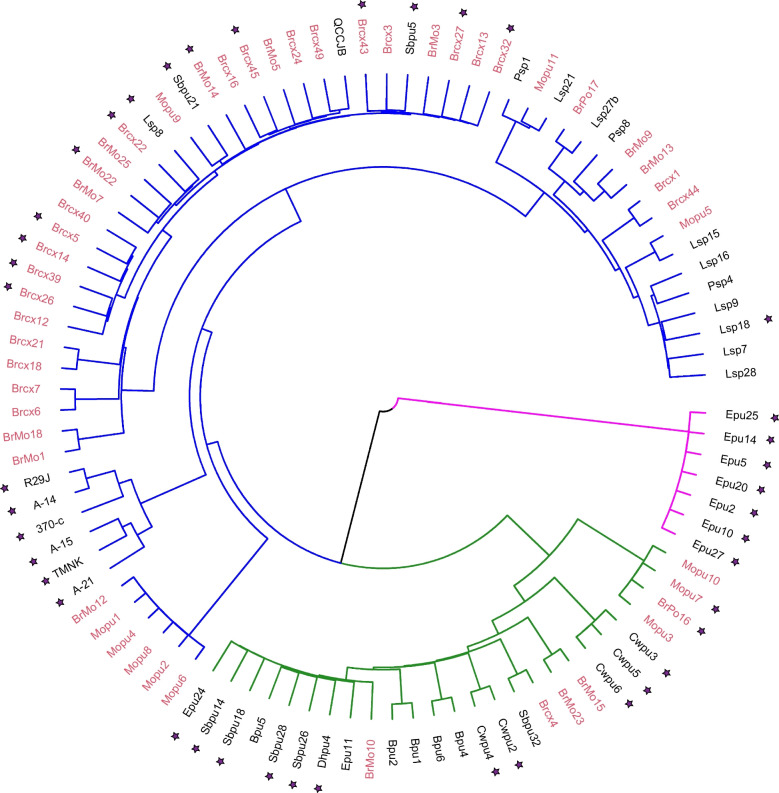
Dendrogram showing genetic relatedness among the *Pgt* isolates (*n* = 96) used in this study. Three major clusters were observed and are indicated by blue, green, and pink colored branches. The first, second, and third cluster includes 63, 26, and 7 *Pgt* isolates, respectively. Red and black labels in the outermost layer represent isolates collected from alternate and cereal hosts, respectively. Isolates avirulent to *Rpg1*_Golden Promise transgenic line H228.2c (GPT) are indicated with a star

Genetic relatedness among the *Pgt* isolates (*n* = 96) was evaluated based on genetic distance using SNP genotype data and visualized with a dendrogram (Fig. 5). Three major clusters were identified within the *Pgt* population based on the lowest BIC value (Fig. 5). The first cluster comprised of 63 *Pgt* isolates including 42 derived from alternate hosts and 21 from cereal hosts (Fig. 5). Twenty-six isolates were grouped into the second cluster comprised of 8 isolates collected from alternate hosts and 18 from cereal hosts (Fig. 5). The third cluster consisted of 7 isolates obtained from cereal hosts only (Fig. 5). The first two clusters were comprised of both virulent and avirulent *Pgt* isolates to the *Rpg1*_trangenic line, H228.2c (*Rpg1* +) (Fig. 5). However, the third cluster included only avirulent isolates to the *Rpg1*_transgenic line H228.2c (Fig. 5). Six Midwest isolates (R29J, A-14, 370-c, A-15, TMNK, and A-21) formed a separate sub-group within the first cluster (Fig. 5). Interestingly, one Midwest isolate (QCCJB) collected from cereal host was more closely related to sexual isolates from the PNW (Fig. 5).

### Linkage disequilibrium (LD)

LD values (*R*^2^) were computed from 1.5 million marker comparisons using 119,595 SNP sites (10% of total SNP markers) generated from the GWS of the 96 *Pgt* isolates. A scatter plot was generated by plotting LD (*R*^2^) values against physical distance (Kb) between marker pairs [34, 35]. The non-linear model [y = log(x)] described the genome-wide LD (*R*2) pattern as shown by the red line in figure 4.7. Based on the model, LD decayed to the *R*^2^ value of 0.3, at physical distance of ~ 10 kb [34, 35]. The extent of LD was detected up to 200 kb [34, 35].

### Association analysis and candidate effector genes

**Fig. 6:**
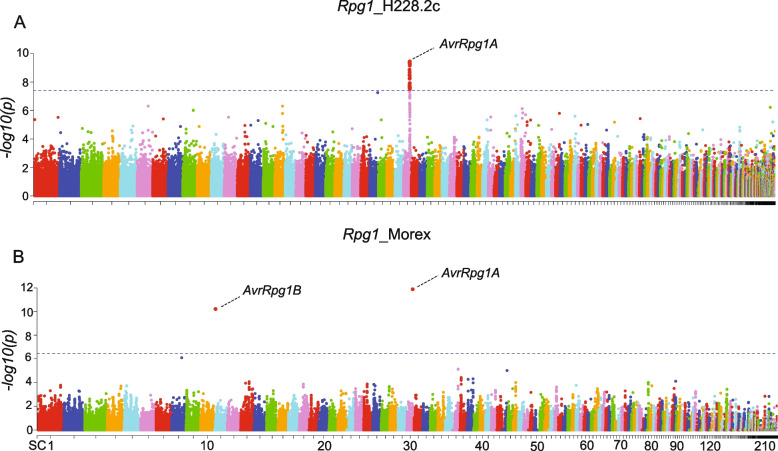
Manhattan plots depicting association analyses results of *Puccinia graminis* f. sp. *tritici* (*Pgt*) isolate phenotypes with two barley lines carrying the *Rpg1* gene; **A** Golden Promise transgenic line (H228.2c), and **B** Morex. X-axis indicates *Pgt* supercontigs. Y-axis represents LOD [-log10(p)] scores for SNP markers. Blue dotted line shows significant threshold of 7.3 LOD value with H228.2c and 6.4 with Morex. *AvrRpg1A* and *AvrRpg1B* are the two avirulence loci corresponding to the barley *R*-gene, *Rpg1*. *AvrRpg1A* was identified with both *Rpg1*_H228.2c and *Rpg1*_Morex. *AvrRpg1B* was identified with *Rpg1*_Morex only

The mixed linear model (MLM) identified 53 significant (*p* < 4.1 × 10^–8^) SNPs associated with disease phenotypes on the *Rpg1*_transgenic line H228.2c (Fig. 6, Table 3). The LOD scores [LOD = -log10(*p*-value)] of significant marker-trait associations (MTAs) extended from 7.5 to 9.4 (Fig. 6). The estimated heritability (*h*^*2*^) of the trait was 0.88. Minor allele frequency (MAF) of the significant SNPs ranged from 0.13 to 0.37 (Table 3). All the significant MTAs corresponded to a single locus, on supercontig2.30 within an ~ 35 kb interval (Supplementary Fig. S1). This locus, designated *AvrRpg1A*, harbored four predicted gene models; *PGTG_10878*, *PGTG_10884*, *PGTG_10885*, and *PGTG_10886* in the delimited 35 kb region. The cumulative phenotypic variance explained by MTAs within *AvrRpg1A* was ~ 23%.

**Table 3:** Significant markers trait associations identified with *Rpg1*_transgenic line H228.2c

Marker	Supercontig	Position^a^	*P*-value^b^	MAF^c^
SSUPERCONT2.30_755426	SUPERCONT2.30	755426	3.56E-10	0.319
SSUPERCONT2.30_756869	SUPERCONT2.30	756869	3.56E-10	0.319
SSUPERCONT2.30_756934	SUPERCONT2.30	756934	3.56E-10	0.319
SSUPERCONT2.30_756920	SUPERCONT2.30	756920	3.56E-10	0.319
SSUPERCONT2.30_756981	SUPERCONT2.30	756981	3.56E-10	0.319
SSUPERCONT2.30_757024	SUPERCONT2.30	757024	3.56E-10	0.319
SSUPERCONT2.30_735285	SUPERCONT2.30	735285	3.71E-10	0.335
SSUPERCONT2.30_735286	SUPERCONT2.30	735286	3.71E-10	0.335
SSUPERCONT2.30_744703	SUPERCONT2.30	744703	3.71E-10	0.335
SSUPERCONT2.30_739727	SUPERCONT2.30	739727	3.71E-10	0.335
SSUPERCONT2.30_742092	SUPERCONT2.30	742092	3.71E-10	0.335
SSUPERCONT2.30_727141	SUPERCONT2.30	727141	4.40E-10	0.330
SSUPERCONT2.30_734539	SUPERCONT2.30	734539	4.40E-10	0.330
SSUPERCONT2.30_726237	SUPERCONT2.30	726237	4.40E-10	0.330
SSUPERCONT2.30_756781	SUPERCONT2.30	756781	4.97E-10	0.266
SSUPERCONT2.30_756838	SUPERCONT2.30	756838	4.97E-10	0.266
SSUPERCONT2.30_757201	SUPERCONT2.30	757201	4.97E-10	0.266
SSUPERCONT2.30_756801	SUPERCONT2.30	756801	5.42E-10	0.314
SSUPERCONT2.30_756905	SUPERCONT2.30	756905	5.43E-10	0.277
SSUPERCONT2.30_756977	SUPERCONT2.30	756977	6.11E-10	0.271
SSUPERCONT2.30_756871	SUPERCONT2.30	756871	6.90E-10	0.303
SSUPERCONT2.30_757158	SUPERCONT2.30	757158	6.90E-10	0.303
SSUPERCONT2.30_757173	SUPERCONT2.30	757173	6.90E-10	0.303
SSUPERCONT2.30_757155	SUPERCONT2.30	757155	6.90E-10	0.303
SSUPERCONT2.30_757172	SUPERCONT2.30	757172	6.90E-10	0.303
SSUPERCONT2.30_756989	SUPERCONT2.30	756989	7.04E-10	0.309
SSUPERCONT2.30_726747	SUPERCONT2.30	726747	1.37E-09	0.324
SSUPERCONT2.30_727118	SUPERCONT2.30	727118	1.37E-09	0.324
SSUPERCONT2.30_727953	SUPERCONT2.30	727953	1.37E-09	0.324
SSUPERCONT2.30_737063	SUPERCONT2.30	737063	2.04E-09	0.213
SSUPERCONT2.30_726793	SUPERCONT2.30	726793	3.04E-09	0.330
SSUPERCONT2.30_738798	SUPERCONT2.30	738798	3.45E-09	0.207
SSUPERCONT2.30_742278	SUPERCONT2.30	742278	3.45E-09	0.207
SSUPERCONT2.30_741612	SUPERCONT2.30	741612	3.45E-09	0.207
SSUPERCONT2.30_757602	SUPERCONT2.30	757602	5.12E-09	0.138
SSUPERCONT2.30_736219	SUPERCONT2.30	736219	5.83E-09	0.202
SSUPERCONT2.30_740039	SUPERCONT2.30	740039	5.83E-09	0.202
SSUPERCONT2.30_751795	SUPERCONT2.30	751795	1.20E-08	0.181
SSUPERCONT2.30_729972	SUPERCONT2.30	729972	1.49E-08	0.207
SSUPERCONT2.30_731095	SUPERCONT2.30	731095	1.49E-08	0.207
SSUPERCONT2.30_726515	SUPERCONT2.30	726515	1.99E-08	0.218
SSUPERCONT2.30_756356	SUPERCONT2.30	756356	2.14E-08	0.154
SSUPERCONT2.30_761120	SUPERCONT2.30	761120	2.32E-08	0.378
SSUPERCONT2.30_761270	SUPERCONT2.30	761270	2.32E-08	0.378
SSUPERCONT2.30_733267	SUPERCONT2.30	733267	2.56E-08	0.207
SSUPERCONT2.30_751829	SUPERCONT2.30	751829	2.96E-08	0.223
SSUPERCONT2.30_751796	SUPERCONT2.30	751796	2.96E-08	0.223
SSUPERCONT2.30_751864	SUPERCONT2.30	751864	2.96E-08	0.223
SSUPERCONT2.30_744521	SUPERCONT2.30	744521	3.00E-08	0.218
SSUPERCONT2.30_760720	SUPERCONT2.30	760720	3.16E-08	0.362
SSUPERCONT2.30_760884	SUPERCONT2.30	760884	3.16E-08	0.362
SSUPERCONT2.30_760856	SUPERCONT2.30	760856	3.16E-08	0.362
SSUPERCONT2.30_760892	SUPERCONT2.30	760892	3.16E-08	0.362

^a^Physical position of marker in bp [50]

^b^Bonferroni corrected threshold *p*-value (< 4.1 × 10^–8^)

^c^Minor allele-frequency

The MLM model did not detect any significant MTAs with the cv Morex infection type data. However, the BLINK model identified two significant (*p* < 3.6 × 10^–7^) MTAs, one on supercontig2.30 and one on supercontig2.11 (Fig. 6, Table 4). The LOD scores of markers located on supercontig2.30 and supercontig2.11 were 11.8 and 10.2, and MAF was 0.32 and 0.47, respectively (Table 4). The MTA on supercontig2.30 and supercontig2.11 explained 7.25 and 1.75% of phenotypic variance, respectively. The estimated heritability (*h*^*2*^) of the trait was determined to be 0.76. The MTA on supercontig2.30 was 55 bp downstream of the predicted gene, *PGTG_10886* within the *AvrRpg1A* locus identified with the *Rpg1*_transgenic line H228.2c infection type data. The MTA on supercontig2.11 was within the predicted gene *PGTG_05433*, and based on flanking non-significant SNPs the locus containing this MTA was delimited to 78 bp. This locus was designated *AvrRpg1B*.

**Table 4:** Significant marker trait associations identified with *Rpg1*_Morex

Marker	Supercontig	Position^a^	*P*-value^b^	MAF^c^
SSUPERCONT2.30_756871	SUPERCONT2.30	756871	1.31E-12	0.324
SSUPERCONT2.11_134838	SUPERCONT2.11	134838	6.21E-11	0.477

^a^Physical position of marker in bp [50]

^b^Bonferroni corrected threshold -value (< 3.6 × 10^–7^)

^c^Minor allele-frequency* p*

### Candidate loci and gene characterization

**Fig. 7:**
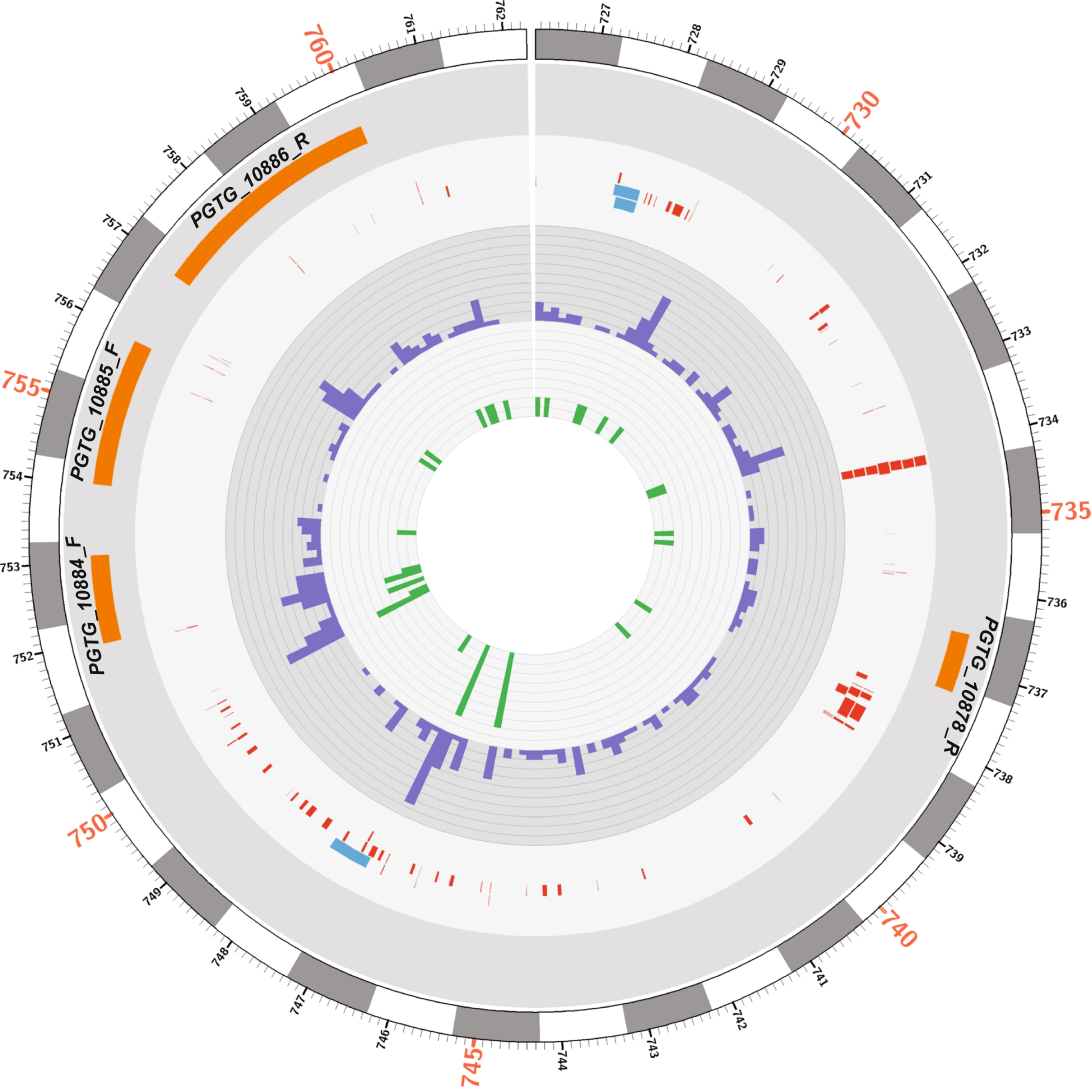
Genome architecture of 35 kb *AvrRpg1A* locus identified with Golden Promise transgenic line (H228.2c). The outer layer shows physical position (kb) on supercontig2.30. Orange tiles indicate the gene space of four candidate genes in the region. Predicted genes are labelled right above tiles along with the orientations (F = forward and R = reverse). Red and blue tiles represent smaller repeats (< 300 bp) and larger repeats (> 300 bp), respectively. Purple bars indicate total SNPs per 200 bp interval. Each axis represents 2 SNPs. Green bars depict total Indels per 200 bp. Two axes depict 1 Indel

The genome architecture of the 35 kb *AvrRpg1A* locus is shown in Fig. 7. Gene density in the genomic region was comparable (4 per 35 kb) to gene density (7 per 35 kb) across the supercontig2.30. The region harbored 102 repeats ranging from 8 to 644 bp, with repeats < 20 bp being the most common. (Fig. 7). SNPs and Indel densities ranged from 0 to 17 and 0 to 4 per 200 bp, respectively (Fig. 7). Candidate effector genes, *PGTG_10878*, *PGTG_10884*, *PGTG_10885*, and *PGTG_10886* encode predicted proteins of 233, 386, 460, and 725 amino acids, respectively (Table 5). PGTG_10878 and PGTG_10884 did not share protein homology with other fungal species and were specific to *Pgt* (Table 5). Protein homologs of PGTG_10885 were found in seven rust species including *Puccinia striiformis* f. sp. *tritici*, *Puccinia triticina*, *Austropuccinia psidii*, *Melampsora americana*, *Melampsora larici-populina*, *Cronartium quercuum* f. sp. *fusiforme*, and *Puccinia sorghi* (Table 5). PGTG_10886 was homologous to proteins of *P. triticina* and *P. striiformis* f. sp. *tritici* (Table 5). A BLAST search of candidate genes against the same reference genome (CRL 75-36-700-3) indicated no duplication events. The CRL 75-36-700-3 reference genome assembly limited the identification of gene space to the supercontig level without anchoring at the chromosome level. Hence, to find the chromosomal location of candidate genes we used the Australian isolate Pgt21-0 with complete chromosomes assembled. BLAST searches of the four *AvrRpg1A* candidate gene sequences against the Pgt21-0 assembly indicated that these genes are located at the telomeric region of chromosome 2 (haplotype B) from 676,683 bp to 700,790 bp.

**Table 5:** Summary of candidate effector gene, encoded protein, and protein homology^a^

Gene	Exons	Nucleotides	Amino acids	Protein	Protein homology
*PGTG_10878*	2	786	233	Hypothetical	none
*PGTG_10884*	1	1161	386	Hypothetical	none
*PGTG_10885*	8	1939	460	26 s protease regulatory subunit 6A	*Puccinia striiformis* f.sp. *tritici*, *Puccinia triticina*, *Austropuccinia psidii*, *Melamspora americana*, *Melamspora larici-populina*, *Cronartium quercuum* f.sp. *fusiforme*, *Puccinia sorghi*
*PGTG_10886*	13	3130	725	Hypothetical	*Puccinia triticina*, *Puccinia striiformis* f.sp*. tritici*
*PGTG_05433*	1	1938	645	Hypothetical	none

^a^Candidate effector genes, *PGTG_10878*, *PGTG_10884*, *PGTG_10885*, and *PGTG_10886* correspond to *Rpg1*_H228.2c. Similarly, candidate genes, *PGTG_10886* and *PGTG_05433* correspond to *Rpg1*_Morex. Gene and protein information is based on the annotation of reference genome of the *Puccinia graminis* f. sp. *tritici* isolate, CRL 75-36-700-3) [50]. Protein homology search was done using blastp tool of NCBI with a threshold set at > 90% coverage and > 90% identity

Two loci were identified with the cv Morex phenotyping data, one colocalizing with the 35 kb *AvrRpg1A* locus on supercontig2.30 and the second delimiting the *AvrRpg1B* locus to a 78 bp region on supercontig2.11. The *AvrRpg1B* locus on supercontig2.11 harbored a single gene, *PGTG_05433*, which was found to be unique to *Pgt* as determined by protein homology searches in the NCBI protein database (Table 5). A local BLAST search of *PGTG_05433* sequence against Pgt21-0 genome assembly revealed three hits on chromosomes 11, 4, and 3 (haplotype B), so a precise chromosomal position could not be determined.

### Significant markers on candidate genes and regulatory regions

**Fig. 8:**
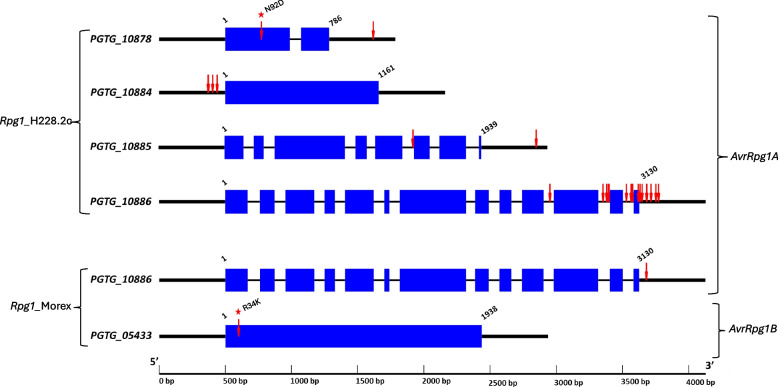
SNP variants in candidate effector genes underlying the *AvrRpg1A* and *AvrRpg1B* loci. For each candidate gene, exons are represented by blue boxes, intron/s by thin black lines, and putative 5’ and 3’ regions (500 bp upstream/downstream) by thick black lines. Red arrows represent SNPs. Red stars show non-synonymous mutations. Four effector gene candidates, *PGTG_10878*, *PGTG_10884*, *PGTG_10885*, and *PGTG_10886* correspond to the *AvrRpg1A* locus and were identified with *Rpg1*_H228.2c disease reactions. For *PGTG_10878*, SNPs are in exon 1 and 3’ region. The single exonic SNP is non-synonymous mutation, N92D. For *PGTG_10884*, SNPs are in the putative promoter region. For *PGTG_10885*, SNPs are in intron 5 and 3’ region. For *PGTG_10886*, SNPs are in intron 10, intron 11, intron 12, exon 13, and 3’ region. The single exonic SNP is a synonymous mutation. Two effector gene candidates, *PGTG_10886* (*AvrRpg1A*) and *PGTG_05433* (*AvrRpg1B*) were identified with the *Rpg1*_Morex disease reactions. For *PGTG_10886*, a single SNP is in 3’ region. For *PGTG_05433*, the single exonic SNP results in the predicted non-synonymous mutation, R34K

Among the 53 MTAs identified with *Rpg1*_transgenic line H228.2c, 26 MTAs were within genic or regulatory regions (500 bp upstream/downstream) of four candidate genes and the remaining 27 markers were located in intergenic regions. Candidate genes *PGTG_10878*, *PGTG_10884*, *PGTG_10885*, and *PGTG_10886* harbored 1, 0, 1 and 11 SNP variants in genic and 1, 4, 1, and 7 variants within putative regulatory regions, respectively (Fig. 8). Among the 13 SNPs in genic regions only two were located in exons, one in the first exon of *PGTG_10878* and another in exon thirteen of *PGTG_10886* (Fig. 8). The exonic SNP in *PGTG_10878* resulted in a predicted nonsynonymous mutation where asparagine was substituted by aspartic acid. The other exonic SNP in *PGTG_10886* was a predicted synonymous mutation (tyrosine to tyrosine).

Among the two MTAs identified with *Rpg1*_Morex, one was within the regulatory region of gene model *PGTG_10886* and another in the exonic region of *PGTG_05433* (Fig. 8). The SNP on *PGTG_05433* caused a predicted nonsynonymous mutation where arginine was replaced by lysine.

### Allele analysis

**Fig. 9:**
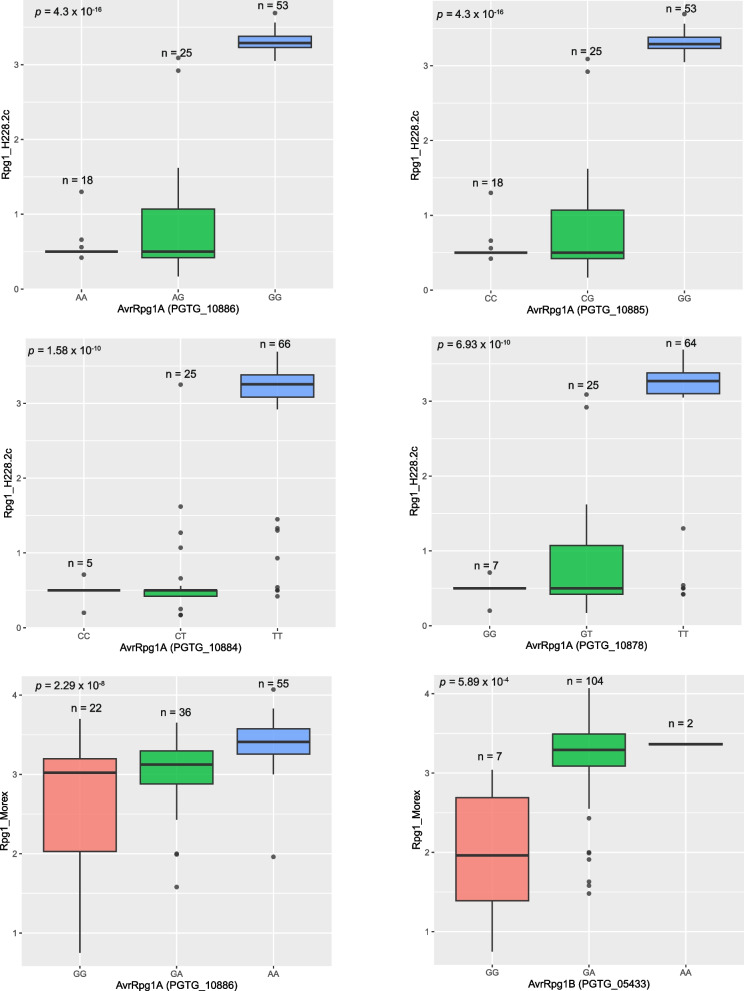
Box plots showing segregation of stem rust phenotypes on barley lines *Rpg1*_H228.2c (Golden Promise transgenic) and *Rpg1*_Morex for *Pgt* isolates with different haplotypes of the most significant SNPs in the *AvrRpg1A* and *AvrRpg1B* candidate genes

The genotypes of significant SNPs identified with *Rpg1*_transgenic line H228.2c were in homozygous or heterozygous state in the majority of avirulent isolates while the alternate homozygous state was identified for virulent isolates (Supplementary Table S3). This indicated that *AvrRpg1A-*mediated avirulence is dominant and virulence is recessive in the *Pgt*/*Rpg1_*H228.2c interaction. Interestingly, the majority of avirulent isolates were in heterozygous state for the significant MTAs (Supplementary Table S3). All eighteen MTAs located within genic or regulatory regions of the gene *PGTG_10886* had near perfect correlations with the phenotypes for the *Pgt* isolates, with a correlation coefficient (|r|) of 0.95 for the most significant SNP in the gene (Supplementary Table S3; Fig. 9). Similarly, all eight MTAs within genic or regulatory regions of the other three candidate genes *PGTG_10878*, *PGTG_10884*, and *PGTG_10885* also had good correlations with observed phenotypes, with the correlation coefficients ranging from 0.72 to 0.95 for the most significant SNP in each gene (Supplementary Table S3; Fig. 9).

For both the significant MTAs identified with *Rpg1*_Morex, the MTA in the regulatory region of the gene model *PGTG_10886* and the MTA within the *PGTG_05433*, moderate correlations (|r|= 0.45 to 0.52) were observed with disease phenotypes (Supplementary Table S4, Fig. 9). The haplotype associated with *PGTG_10886* was in heterozygous or homozygous state in 20 avirulent isolates and in alternate homozygous form in 53 virulent isolates (Supplementary Table S4; Fig. 9). These data suggest that avirulence is dominant and virulence is recessive in the *Pgt*/*Rpg1*_Morex interaction as was determined for the *Pgt*/*Rpg1_*H228.2c interaction.

## Discussion

The first North American isolate collected from barley, designated *Pgt* race QCCJB based on wheat stem rust differential lines, identified as virulent on barley lines containing *Rpg1* was hypothesized to have originated from the Pacific Northwest (PNW) population [12]. Interestingly, our recent virulence profiling of *Pgt* isolates collected from barley in eastern Washington showed that *Rpg1* virulence was predominate (99%) in this *Pgt* population [15]. This suggests that evolutionary pressure on this population selected for this virulence, however this is perplexing considering that *Rpg1* had not been deployed in PNW barley varieties. To identify *Pgt* virulence/avirulence loci that evolved to overcome *Rpg1*-mediated stem rust resistance in barley, we utilized whole genome shotgun sequencing, and phenotyping data on two barley lines containing *Rpg1* for these diverse isolates collected from the PNW sexual population and isolates collected from the Midwestern US asexual population for genome wide association studies (GWAS). Two loci corresponding to *Rpg1* avirulence were identified. The major locus, *AvrRpg1A* (accounted for ~ 23% estimated phenotypic variance) mapped to a 35 kb interval on supercontig2.30 that contains four candidate effector genes. The minor locus, *AvrRpg1B* landed on a single gene within a 78 bp region on supercontig2.11. The detection of only two dominant avirulence loci that interact with *Rpg1* indicated typical gene-for-gene interactions similar to those characterized for other rust pathosystems [63, 64].

*In planta* transcriptome data has been commonly used to identify candidate effector genes in several fungal pathogens including rust [22]. One prominent limitation of transcriptomics is difficulty to identify major loci contributing to disease phenotype since several to hundreds of genes are temporally expressed during the infection process [30, 50]. Bi-parental mapping is another powerful genetic tool that has been used to map virulence/avirulence loci in the rust pathogen, *P. striiformis* f. sp. *tritici* (*Pst*), a close relative of *Puccinia graminis* f. sp. *tritici* (*Pgt*) [65–67]. Unlike *Pst*, creating a crossing population is challenging for *Pgt* because *Pgt* teliospores exhibit a very strong dormancy for an extended period [68]. To overcome these limitations with transcriptomics and bi-parental mapping, we utilized genome wide association studies (GWAS) to map avirulence loci corresponding to the barley *Rpg1* gene. The benefit of GWAS is that it can utilize population scale variability to identify significant marker-trait associations (MTAs) linked to trait of interest [69]. The power of GWAS to detect robust associations depends upon several factors including population size, type, phenotype and genotype data, and statistical tools used for association analysis [70].

The *Pgt* isolates utilized in this study represent a suitable population size to map virulence/avirulence loci in the *Pgt* genome (~ 89 Mb). Several avirulence genes were mapped across the *Pst* genome utilizing much smaller populations of 14 and 30 *Pst* individuals [71, 72]. In a recent review, a population size of 50–100 diverse individuals was considered adequate to precisely identify MTAs in pathogens [73]. The type of population, sexual or asexual, can significantly influence the resolution of genome-wide scans [73]. The *Pgt* population in this study comprised of a majority of isolates (*n* = 89) collected from the states of Washington and Idaho. The PNW region of North America (US states Washington, Idaho, and Oregon and Canadian province British Columbia) is recognized to harbor sexual population of *Pgt* with a high level of virulence diversity [11, 13]. In a recent rust survey around the Washington-Idaho border, as many as 16 *Pgt* races were detected in a single field [74]. Roelfs and Groth [11] identified > 100 races among 426 *Pgt* isolates assayed from the PNW region. Higher meiotic recombination rates in the genomes of sexual populations break linkage blocks which allow for higher resolution mapping of virulence/avirulence loci utilizing GWAS [73]. The linkage decay (LD) in the *Pgt* population utilized in this study was approximately 10 kb across the genome, suggesting a sexual population with a high level of recombination [34, 35]. Since the collapsed haploid genome assembly was used for genomic analysis, including LD computation, the LD value reported here might have been underestimated to a certain degree; however, lower LD value would be expected in a population dominated by sexually originated isolates.

Robust disease phenotype data was generated for 96 and 113 *Pgt* isolates on two sources of the *Rpg1* gene, Golden Promise transgenic line H228.2c and cv Morex, respectively, by performing disease assays under temperature and light-controlled conditions. Virulence profiles were determined based on infection types and the *Pgt* population showed a bimodal phenotypic distribution on H228.2c with an approximately 40:60 ratio of avirulent to virulent isolates (Fig. 1). The distinct segregation for contrasting phenotypes shows that this population is suitable for mapping avirulence/virulence loci corresponding to the *Rpg1* gene in the H228.2c transgenic line. Avirulence/virulence on cv Morex was skewed where 17% of the isolates were avirulent and 83% were virulent out of 113 isolates used which included both the PNW population and Midwestern isolates. Although H228.2c carries a single copy of *Rpg1* from cv Morex in the Golden Promise background, it was shown in previous studies [15, 41] and in this study (Fig. 1, Supplementary Table S1) to contain an elevated level of resistance to isolates avirulent on *Rpg1* compared to cv Morex. Also, a larger proportion of the isolates were avirulent on H228.2c (43%) compared to the isolates avirulent on cv Morex (17%). The level of resistance on the avirulent isolates for H228.2c is near immunity (IT range of 0 to 2 and mode of 0;) whereas cv Morex provided a range from resistant to moderately resistant (IT range of 0; to 23- and mode of 21) to the avirulent isolates. Also, all the isolates avirulent on cv Morex showed near immunity reactions on H228.2c. We hypothesize that the resistance response mediated by the *AvrRpg1*- *Rpg1* gene-for-gene interaction is enhanced by a genetic component in the cv Golden Promise background, or some isolates contain a suppressor of resistance that functions in the cv Morex interaction but does not function in the Golden Promise transgenic line H228.2c. Since the PNW population (*n* = 89) has a low percentage of isolates avirulent on *Rpg1* in the cv Morex background (10%) the phenotype data previously generated for 17 Midwestern US isolates [30] were added to the panel of the 96 *Pgt* isolates (PNW = 89, MW = 7) to increase the power of avirulence/virulence gene mapping with cv Morex.

Genotyping technologies like GBS or RNA sequencing are cheaper due to the reduced representation but present limitations to high density genotyping. One pitfall of GBS is uneven sequencing coverage resulting in a high proportion of missing data [75]. RNAseq on the other hand only detects sequence variation within expressed genes [76]. Due to these constraints of GBS and RNAseq, a whole genome shotgun sequencing (WGS) approach was utilized to genotype the *Pgt* population. One major benefit of WGS is that it allows for the detection of variation in both gene coding and non-coding regions [77]. For precise variant detection high coverage sequence data is essential [77], thus 96 *Pgt* isolates were sequenced to an average depth of 113 × generating sequencing reads with Q30 for 94% of the bases. This high-quality sequencing data was suitable for variant calling and downstream processing. We obtained a total of 1,195,947 SNPs and 168,516 Indels for the 96 isolates. This corresponds to 13 SNPs and 2 Indels per kilobase of the *Pgt* genome (~ 89 Mbp) indicating high marker density for this natural *Pgt* population. This marker density and population size was much more robust than a recent study where 30 *Pst* isolates were sequenced to 30 × depth for the purpose of association mapping [72]. Of the > 1 million variants identified in this *Pgt* population deleterious variants included splice, start, stop lost/gained, splice, missense, frameshift, conservation inframe deletion/insertion, and disruptive inframe deletion/insertion variants that accounted for 2.9 and 2.8% of total SNPs and Indel effects, respectively.

The level of genome homozygosity/heterozygosity is often used as an indicator of the mode of reproduction in an organism [78–80]. In diploid or binuclear fungi asexual reproduction is expected to increase genome heterozygosity due to accumulation of distinct mutations in one genome copy that are unable to recombine to form homozygotes [78]. Based on the rates of SNP homozygosity of isolates collected from the primary cereal hosts barley and wheat, it can be inferred that sexual reproduction is the major means of propagation for *Pgt* in the PNW region. However, a comparatively higher level of heterozygosity of seven cereal isolates indicated that PNW *Pgt* also reproduces asexually. Apart from a few exceptions, the rate of SNP homozygosity in isolates collected from cereal (barley and wheat) and alternate hosts (Mahonia and barberry) were similar. This indicated that sexual hosts are a major source of inoculum on PNW cereals. Originally, we hypothesized that six of seven isolates collected from the Midwest US are of asexual origin so should carry highly heterozygous genomes. To our surprise, the heterozygosity rate (37–44%) for these six isolates was comparable to average heterozygosity (35%) of isolates from the alternate hosts. This indicates that Midwest isolates utilized in this study may have undergone a shorter asexual reproduction period than previously expected resulting in the accumulation of fewer mutations present in the heterozygous state. This data doesn’t fit with the hypothesis that these isolates were collected from a stabilized asexual population as they were collected after sexual hosts were eradicated from the Midwestern US. The midwestern isolates utilized in this study were collected from 1977 to 1999, after barberry eradication phased out in the late 1970s. The virulence profile of Midwest isolate, QCCJB resembles sexual isolates from the PNW, so it was speculated that QCCJB probably originated from the PNW [12]. The WGS data generated was utilized to provide conclusive evidence that QCCJB did originate from the PNW region and was disseminated eastward into the Midwest. The level of genome homozygosity (63%) supports the sexual origin of QCCJB. Based on hierarchical clustering (Fig. 5), QCCJB is more closely related to PNW isolates collected from the alternate host barberry while the other six Midwestern isolates formed a separate sub-group within the same cluster. Initially we hypothesized that the clustering of isolates would be based on the host from which they were derived. However, two major clusters included isolates originated from both primary cereal hosts barley and wheat and alternate sexual hosts barberry and Mahonia. Interestingly, cereal isolates were clustered based on geographic location. For example, cereal isolates from the Valley ford, WA region formed close sub-groups within the first cluster and the majority of cereal isolates from the Pullman, WA region were in second and third cluster. However, sexual isolates had a lower degree of sub-grouping based on the location of collection.

Association mapping identified 53 and 2 significant SNPs associated with disease phenotype on the Golden Promise transgenic line H228.2c and cv Morex, respectively. One major pitfall of GWAS is spurious association of genotype and phenotype resulting in detection of false-positive associations [81]. We tried to minimize this error by incorporating structure (Q) and relatedness (K) data as covariate in the MLM model with H228.2c and structure (Q) in the BLINK model with cv Morex. Also, to alleviate the possibility of false associations stringent thresholds of 7.3 and 6.4 LOD scores were used with H228.2c and cv Morex, respectively. Two novel QTLs were identified for *Avr_Rpg1*, one on supercontig2.30 (designated *Avr_Rpg1A)* and one on supercontig2.11 (designated *Avr_Rpg1B)*. The major avirulence locus on supercontig2.30 represents a common locus identified for both H228.2c and cv Morex. Haplotype analysis of significant SNPs at this locus indicated that the majority of avirulent isolates share common haplotypes in the homozygous or heterozygous state whereas the virulent isolates share the alternate putative recessive virulent haplotype in the homozygous state. This suggests that this locus harbors a dominant avirulence gene shared by the majority of avirulent isolates hence is considered the major *Avr_Rpg1* locus. The *Avr_Rpg1B* locus identified on supercontig2.11 with cv Morex was considered the minor locus because several avirulent isolates carry the same allele present among virulent isolates. This indicates that only a few avirulent isolates carry this avirulence effector or possibly represents a false positive association.

Harold Henry Flor in his landmark paper described the gene-for-gene hypothesis which explained the dominant nature of pathogen avirulence genes and their specific interactions with dominant host resistance genes on the basis of specific genetic interaction of flax rust, *Melamspora lini* with its host, flax (*Linum usitatissimum*) [82]. Based on the classical gene-for-gene hypothesis, the product of the dominant avirulence gene is directly or indirectly recognized by the dominant R-gene protein, resulting in resistance or an incompatible disease reaction [83]. Surprisingly, in the wheat-stripe rust pathosystem, *P. striiformis* f. sp. *tritici* (*Pst*) avirulence corresponding to several wheat stripe rust R-genes were determined to be recessive in contrast to the well-established dogma of dominant avirulence and recessive virulence in host-pathogen genetic interaction with biotrophic pathogens [65, 66]. However, here we determined *Pgt* avirulence to *Rpg1* is dominant based on the state of haplotypes (heterozygous/homozygous) carried by avirulent isolates at the major *Avr_Rpg1A* locus. Interestingly, the dominant avirulence haplotype was present in the heterozygous state for the majority of the avirulent isolates. Two cloned *Pgt* effector genes, *AvrSr35* and *Avr50* were also present in heterozygous state in the mutants from which they were identified [23, 24]. Mutation of *Avr_Rpg1* could lead to loss of recognition by *Rpg1*, and the evolution of *Rpg1* virulence in the PNW population. This hypothesis is supported by primary amino acid substitutions, N92D and R34K in two candidate effectors, PGTG_10878 and PGTG_05433, respectively. Host selection pressure is considered crucial for the evolution of virulence on deployed resistance genes in pathogen populations [84]. Thus, it is quite surprising that the PNW *Pgt* population assayed is dominated by isolates virulent on *Rpg1* considering that the R-gene has not been deployed in commercial cultivars grown in the region. Previous studies have reported several wild grasses like *Elymus glaucus*, *Agrostis alba*, *Elytrigia repens*, and *Elymus canadensis* host a native *Pgt* population in the PNW [13]. It is possible that these wild grasses or others carry resistance mechanisms similar to *Rpg1*-mediated resistance that exerted selection pressure to overcome *Rpg1* or there is a similar unknown resistance mechanism in wheat or the alternate sexual hosts that are applying the selection pressure for isolates virulent on *Rpg1*. To begin answering these questions of *Rpg1* virulence evolution and mechanisms, it is essential to identify the Avr-Rpg1 effector or virulence effectors that suppress *Rpg1*-mediated resistance.

The major *Avr_Rpg1A* locus on supercontig 2.30 harbors a total of four candidate genes (*PGTG_10878*, *PGTG_10884*, *PGTG_10885*, and *PGTG_10886*) within a 35 kb interval and the minor *Avr_Rpg1B* locus contains a single candidate gene (*PGTG_05433*) within a 78 bp interval on supercontig2.11. Repeats identified within the major *Avr_Rpg1A* locus on supercontig 2.30 were mostly small (< 20 bp) and represented a small portion (11%) of the region (Fig. 7). Also, the gene density in this region was comparable to rest of supecontig2.30. In several fungi including *Fusarium oxysporum* f sp. *lycopersici*, *Leptosphaeria maculans*, and *Zymoseptoria tritici* effector genes were reported in gene poor and repeat rich regions of the genome [73]. However, genomes of rust fungi including *Pgt* and *Pst* do not follow the two-speed genome model [50, 85]. Among the candidate genes, *PGTG_10878* encodes a small protein (< 300 aa), *PGTG_10884* and *PGTG_10885* encode medium sized proteins (300–500 aa) typical of known secreted effectors whereas *PGTG_10886* and *PGTG_05433* encode larger proteins (> 500 aa). Several cloned *Pgt* effector genes including *AvrSr35* (578 aa) [23], *RGD* (818 aa) [21], and *VPS9* (744 aa) [21] encode larger proteins while *AvrSr50* (132 aa) [24] and *AvrSr27* (144 aa) [25] encode small proteins. Hence, protein size alone cannot be used to prioritize candidate effector genes in *Pgt*. Three candidate genes were unique to *Pgt* suggesting that they either evolved de novo or were acquired horizontally. Two genes were probably acquired from ancestral rust during speciation since they shared sequence similarity with other rust species. It is important to note that all effector genes are not species specific, and some are shared by several fungal species. For example, *Verticillium dahliae* avirulence effector, *Ave1* that interacts with *Ve1* in tomato has homology or orthologs in many fungal species and bacteria [86].

Effectors are pathogen molecules that evolve virulence function to successfully colonize their host by suppressing plant defense response [87] or evolve to aid in nutrient acquisition [88]. These effector molecules when detected by host R-gene receptors, cytoplasmic or intracellular, become avirulence effectors as they elicit the plant resistance responses [89]. However, avirulence effectors can escape R-gene mediated recognition through molecular arms race evolutionary processes as depicted by the zig-zag model proposed by [90]. Avirulence genes can be disrupted through mechanisms including insertion, deletion, and nucleotide substitutions resulting in differential expression, amino acid sequence alteration and protein modification [73]. Here, we show that nucleotide polymorphisms within the primary coding sequences or within regulatory regions of the candidate *Avr_Rpg1A* and *Avr_Rpg1B* avirulence genes delimited by our GWAS analysis potentially leads to loss of avirulent effector recognition by the cognate *Rpg1* receptor. Since we identified candidate effector genes based on the result of genotype and phenotype association, the top candidate gene should be the one with a high degree of correlation between allele type and observed phenotypes. Based on this criterion, the gene model *PGTG_10886* is the top candidate *Avr*_*Rpg1A* gene. Surprisingly, polymorphism detected on *PGTG_10886* between virulent and avirulent isolates were in introns, near splice sites and 3’-regulatory region, except a SNP in exon 13 that caused a synonymous mutation (Fig. 8). Hence, functional studies will be crucial to validate our candidate genes and elucidate the role of the causal mutations in phenotypic diversity. Also, it is important to note that *Pgt* gene architectures are based on predictions with computer algorithms, and thus misannotation can result in incorrect predictions of causal mutations [50]. Another candidate gene, *PGTG_10878* identified within the *AvrRpg1A* locus, contains a non-synonymous mutation (N92D) which is highly correlated (~ 90%) with disease phenotype of virulent and avirulent isolates (Supplementary Table S3). Furthermore, the gene is unique to *Pgt* and encodes a small protein. Hence, *PGTG_10878* is also a strong *Avr_Rpg1A* candidate gene. The candidate gene, *PGTG_05433* at the minor *Avr_Rpg1*B locus also harbors a single nucleotide variant in its coding sequence that is predicted to cause the non-synonymous mutation, arginine to lysine at amino acid position 34. However, genotype by phenotype correlation was not strong; therefore, the Avr_Rpg1B effector is probably shared by a low proportion of avirulent isolates or this locus possibly represents a false association. Thus, two strong *Avr_Rpg1A* candidate genes, *PGTG_10886* and *PGTG_10978* will be prioritized for initial functional validation work.

## Conclusions

It is crucial to understand the evolutionary mechanism of virulence on important *R*-genes which is especially important in the barley-*Pgt* pathosystem where the primary germplasm pool only contains three characterized effective stem rust resistance genes, *Rpg1*, *RMRL*, and *Rpg7*. Thus, to understand effective deployment of resistances the identification of avirulence loci/genes is the first step towards unravelling the evolutionary forces that enable effectors to evade or suppress R-gene mediated defense signaling. We utilized ~ 1.2 million SNPs to map avirulence loci in *Pgt* via a GWAS approach. A total of 55 MTAs were identified corresponding to two unique loci associated with *AvrRpg1* that interact with the barley *Rpg1* gene. The major *Avr_Rpg1A* locus harbored four candidate effector genes and the minor *Avr_Rpg1B* locus harbored one candidate gene. The dominant nature of the *Avr_Rpg1A* locus agrees with Flor’s classical gene-for-gene model. Several markers identified in this study perfectly correlated with pathogen phenotype, hence, they can be utilized to screen *Pgt* population for *Rpg1* virulence. The candidate *Avr_Rpg1A* and *Avr_Rpg1B* genes identified in this study will be utilized for future functional validation work. Thus, this study begins to fill gaps in our current understanding of host-pathogen interaction in the barley-*Pgt* pathosystem.

### Supplementary Information

Supplementary Material 1: Supplementary Table S1. Information on collection year, location, and host of the *Pgt* isolates used in this study.

Supplementary Material 2: Supplementary Table S2. Stem rust disease phenotype of the *Pgt* isolates on barley lines Steptoe, Harrington, Golden Promise, Morex, and Golden Promise transgenic (GPT).

Supplementary Material 3: Supplementary Table S3. Haplotypes of marker-trait associations (MTAs) identified with Golden Promise transgenic line (H228.2c) carrying the *Rpg1* gene.

Supplementary Material 4: Supplementary Table S4. Haplotypes of marker-trait associations (MTAs) identified with the barley line Morex carrying the *Rpg1* gene.

Supplementary Material 5: Supplementary Fig. S1. *AvrRpg1A* locus identified with Golden promise transgenic line (H228.2c) delimited to 35 kb interval on supercontig2.30. X-axis represents physical position on supercontig2.30. Y-axis represents LOD scores of SNP markers. Blue dotted line represents the significance threshold (LOD = 7.3). Black dotted lines indicate 35 kb interval corresponding to significant markers.

Supplementary Material 6: Supplementary Fig. S2. PCA plots depicting the structure of *Pgt* population (*n* = 96). A] PC1 plotted against PC2 B] PC2 plotted against PC3. The percentage of variance explained by PC1, PC2, and PC3 was 15.60%, 6.35%, and 4.55%, respectively. Pink and blue dots indicate isolates collected from cereal and alternate hosts, respectively.

## Data Availability

All datasets generated in the present study are included in this published article and its supplementary files. The sequencing read data are available in the NCBI’s Sequence Read Archive (SRA) database, under the Bio project accession ID PRJNA952228 (https://www.ncbi.nlm.nih.gov/bioproject/952228).

## References

[1] Roelfs AP, Singh RP, Saari EE. Rust diseases of wheat: concepts and methods of disease management. Mexico: CIMMYT; 1992.

[2] Singh RP, Hodson DP, Jin Y, Lagudah ES, Ayliffe MA, Bhavani S, et al. Emergence and spread of new races of wheat stem rust fungus: continued threat to food security and prospects of genetic control. Phytopathology. 2015;105:872–84. 26120730 10.1094/PHYTO-01-15-0030-FI

[3] Roelfs AP, United States Agricultural Research Service. Estimated Losses Caused by Rust in Small Grain Cereals in the United States, 1918-76. Vol. 1356-1372. Department of Agriculture, Agricultural Research Service; 1978. https://books.google.com/books?id=AlsvAAAAYAAJ.

[4] Roelfs AP. Epidemiology in North America. In: Roelfs AP, Bushnell WR, editors. The cereal rusts, vol. 2, diseases, distribution, epidemiology, and control. Orlando: Academic; 1985. p. 403–34.

[5] Steffenson BJ. Analysis of durable resistance to stem rust in barley. Euphytica. 1992;63:153–67. 10.1007/BF00023920

[6] Roelfs AP. Effects of Barberry eradication. Plant Dis. 1982;66:177–81. 10.1094/PD-66-177 30786626

[7] Pretorius ZA, Singh RP, Wagoire WW, Payne TS. Detection of virulence to wheat stem rust resistance gene *Sr31* in *Puccinia graminis*. f. sp. *tritici* in Uganda. Plant Dis. 2000;84:203–203. 30841334 10.1094/PDIS.2000.84.2.203B

[8] Berlin A. Stem rust attacks in Sweden heralds the return of a previously vanquished foe. 2017. https://www.slu.se/en/ew-news/2017/11/stem-rust-attacks-in-sweden-heralds-the-return-of-a-previously-vanquished-foe/#:~:text=Stem%20rust%20or%20black%20rust,have%20returned%20to%20the%20north. Accessed 5 Apr 2023.

[9] Lewis CM, Persoons A, Bebber DP, Kigathi RN, Maintz J, Findlay K, et al. Potential for re-emergence of wheat stem rust in the United Kingdom. Commun Biol. 2018;1:13. 30271900 10.1038/s42003-018-0013-y PMC6053080

[10] Saunders DG, Pretorius ZA, Hovmøller MS. Tackling the re-emergence of wheat stem rust in Western Europe. Commun Biol. 2019;2:51. 30729187 10.1038/s42003-019-0294-9 PMC6361993

[11] Roelfs AP, Groth JV. A comparison of virulence phenotypes in wheat stem rust populations reproducing sexually and asexually. Phytopathology. 1980;70:855–62. 10.1094/Phyto-70-855

[12] Jin Y. Role of *Berberis* spp. as alternate hosts in generating new races of *Puccinia graminis* and *P. **striiformis*. Euphytica. 2011;179:105–8. 10.1007/s10681-010-0328-3

[13] Jin Y, Rouse M, Groth J. Population diversity of *Puccinia graminis* is sustained through sexual cycle on alternate hosts. J Integr Agric. 2014;13:262–4. 10.1016/S2095-3119(13)60647-4

[14] Wang MN, Wan AM, Chen XM. Barberry as alternate host is important for Puccinia graminis f. sp. tritici but not for *Puccinia striiformis* f. sp. *tritici* in the US Pacific Northwest. Plant Dis. 2015;99:1507–16. 30695965 10.1094/PDIS-12-14-1279-RE

[15] Upadhaya A, Upadhaya SG, Brueggeman R. The wheat stem rust (*Puccinia graminis* f. sp. tritici) population from Washington contains the most virulent isolates reported on barley. Plant Dis. 2022;106:223–30. 34546770 10.1094/PDIS-06-21-1195-RE

[16] Brueggeman R, Rostoks N, Kudrna D, Kilian A, Han F, Chen J, et al. The barley stem rust-resistance gene *Rpg1* is a novel disease-resistance gene with homology to receptor kinases. Proc Natl Acad Sci. 2002;99:9328–33. 12077318 10.1073/pnas.142284999 PMC123140

[17] Brueggeman R, Druka A, Nirmala J, Cavileer T, Drader T, Rostoks N, et al. The stem rust resistance gene *Rpg5* encodes a protein with nucleotide-binding-site, leucine-rich, and protein kinase domains. Proc Natl Acad Sci. 2008;105:14970–5. 18812501 10.1073/pnas.0807270105 PMC2567477

[18] Henningsen E, Sallam AH, Matny O, Szinyei T, Figueroa M, Steffenson BJ. Rpg7: a new gene for stem rust resistance from *Hordeum** vulgare* ssp. spontaneum. Phytopathology. 2021;111:548–58. 32880513 10.1094/PHYTO-08-20-0325-R

[19] Kilian A, Steffenson BJ, Maroof S, Kleinhofs A. RFLP markers linked to the durable stem rust resistance gene *Rpg1* in barley. Mol Plant Microbe Interact. 1994;7:298–301. 7912120 10.1094/MPMI-7-0298

[20] Nirmala J, Drader T, Chen X, Steffenson B, Kleinhofs A. Stem rust spores elicit rapid RPG1 phosphorylation. Mol Plant Microbe Interact. 2010;23:1635–42. 20653415 10.1094/MPMI-06-10-0136

[21] Nirmala J, Drader T, Lawrence PK, Yin C, Hulbert S, Steber CM, et al. Concerted action of two avirulent spore effectors activates *Reaction to Puccinia graminis 1* (*Rpg1*)-mediated cereal stem rust resistance. Proc Natl Acad Sci. 2011;108:14676–81. 21873196 10.1073/pnas.1111771108 PMC3167542

[22] Lorrain C, Gonçalves dos Santos KC, Germain H, Hecker A, Duplessis S. Advances in understanding obligate biotrophy in rust fungi. New Phytol. 2019;222:1190–206. 30554421 10.1111/nph.15641

[23] Salcedo A, Rutter W, Wang S, Akhunova A, Bolus S, Chao S, et al. Variation in the *AvrSr35* gene determines *Sr35* resistance against wheat stem rust race Ug99. Science. 2017;358:1604–6. 29269474 10.1126/science.aao7294 PMC6518949

[24] Chen J, Upadhyaya NM, Ortiz D, Sperschneider J, Li F, Bouton C, et al. Loss of *AvrSr50* by somatic exchange in stem rust leads to virulence for *Sr50* resistance in wheat. Science. 2017;358:1607–10. 29269475 10.1126/science.aao4810

[25] Upadhyaya NM, Mago R, Panwar V, Hewitt T, Luo M, Chen J, et al. Genomics accelerated isolation of a new stem rust avirulence gene–wheat resistance gene pair. Nat Plants. 2021;7:1220–8. 34294906 10.1038/s41477-021-00971-5

[26] Arndell T, Chen J, Sperschneider J, Upadhyaya NM, Blundell C, Niesner N, et al. Pooled effector library screening in protoplasts rapidly identifies novel *Avr* genes. Nat Plants. 2024;10:572–80. 38409291 10.1038/s41477-024-01641-y PMC11035141

[27] Shjerve RA, Faris JD, Brueggeman RS, Yan C, Zhu Y, Koladia V, et al. Evaluation of a *Pyrenophora teres* f. *teres* mapping population reveals multiple independent interactions with a region of barley chromosome 6H. Fungal Genet Biol. 2014;70:104–12. 25093269 10.1016/j.fgb.2014.07.012

[28] Upadhyaya NM, Garnica DP, Karaoglu H, Sperschneider J, Nemri A, Xu B, et al. Comparative genomics of Australian isolates of the wheat stem rust pathogen *Puccinia graminis* f. sp. *tritici* reveals extensive polymorphism in candidate effector genes. Front Plant Sci. 2015;5:759. 25620970 10.3389/fpls.2014.00759 PMC4288056

[29] Carlsen SA, Neupane A, Wyatt NA, Richards JK, Faris JD, Xu SS, et al. Characterizing the *Pyrenophora teres* f. *maculata*–barley interaction using pathogen genetics. G3. 2017;7:2615–26. 28659291 10.1534/g3.117.043265 PMC5555467

[30] Sharma Poudel R, Richards J, Shrestha S, Solanki S, Brueggeman R. Transcriptome-wide association study identifies putative elicitors/suppressor of *Puccinia graminis* f. sp. *tritici* that modulate barley rpg4-mediated stem rust resistance. BMC Genomics. 2019;20:1–21. 31842749 10.1186/s12864-019-6369-7 PMC6915985

[31] Hartmann FE, Sánchez-Vallet A, McDonald BA, Croll D. A fungal wheat pathogen evolved host specialization by extensive chromosomal rearrangements. ISME. 2017;11:1189–204. 10.1038/ismej.2016.196 PMC5437930 28117833

[32] Richards JK, Stukenbrock EH, Carpenter J, Liu Z, Cowger C, Faris JD, et al. Local adaptation drives the diversification of effectors in the fungal wheat pathogen *Parastagonospora nodorum* in the United States. PLoS Genet. 2019;15:e1008223. 31626626 10.1371/journal.pgen.1008223 PMC6821140

[33] Richards JK, Kariyawasam GK, Seneviratne S, Wyatt NA, Xu SS, Liu Z, et al. Triple threat: the *Parastagonospora nodorum* SnTox267 effector exploits three distinct host genetic factors to cause disease in wheat. New Phytol. 2022;233:427–42. 34227112 10.1111/nph.17601 PMC9292537

[34] Upadhaya A. Genetic characterization of virulence in a Pacific Northwest stem rust population and mapping of new sources of resistance in barley. PhD dissertation. Washington State University; 2023. 10.7273/000005216.

[35] Upadhaya A, Upadhaya SG, Brueggeman R. Identification of candidate avirulence and virulence genes corresponding to stem rust (*Puccinia graminis* f. sp. *tritici*) resistance genes in wheat. Mol Plant Microbe Interact. 2024. 10.1094/MPMI-05-24-0056-R. 38780476 10.1094/MPMI-05-24-0056-R

[36] Tibbs Cortes L, Zhang Z, Yu J. Status and prospects of genome-wide association studies in plants. Plant Genom. 2021;14:e20077. 10.1002/tpg2.20077 PMC12806871 33442955

[37] Gao Y, Liu Z, Faris JD, Richards J, Brueggeman RS, Li X, et al. Validation of genome-wide association studies as a tool to identify virulence factors in *Parastagonospora nodorum*. Phytopathology. 2016;106:1177–85. 27442533 10.1094/PHYTO-02-16-0113-FI

[38] Clare SJ, Duellman KM, Richards JK, Poudel RS, Merrick LF, Friesen TL, et al. Association mapping reveals a reciprocal virulence/avirulence locus within diverse US *Pyrenophora teres* f. *maculata* isolates. BMC Genomics. 2022;23:1–17. 35397514 10.1186/s12864-022-08529-1 PMC8994276

[39] Vleeshouwers VG, Oliver RP. Effectors as tools in disease resistance breeding against biotrophic, hemibiotrophic, and necrotrophic plant pathogens. Mol Plant Microbe Interact. 2014;27:196–206. 24405032 10.1094/MPMI-10-13-0313-IA

[40] Li F, Upadhyaya NM, Sperschneider J, Matny O, Nguyen-Phuc H, Mago R, et al. Emergence of the Ug99 lineage of the wheat stem rust pathogen through somatic hybridization. Nat Commun. 2019;10:5068. 31699975 10.1038/s41467-019-12927-7 PMC6838127

[41] Horvath H, Rostoks N, Brueggeman R, Steffenson B, Von Wettstein D, Kleinhofs A. Genetically engineered stem rust resistance in barley using the *Rpg1* gene. Proc Natl Acad Sci. 2003;100:364–9. 12509512 10.1073/pnas.0136911100 PMC140979

[42] Rasmusson DC, Wilcoxson RD. Registration of “Morex” Barley. Crop Sci. 1979;19:293. 10.2135/cropsci1979.0011183X001900020032x.

[43] Muir CE, Nilan RA. Registration of Steptoe Barley 1 (Reg. No. 134). Crop Sci. 1973;13:770–770. 10.2135/cropsci1973.0011183X001300060063x

[44] Harvey BL, Rossnagel BG. Harrington barley. Can J Plant Sci. 1984;64:193–4. 10.4141/cjps84-024

[45] Stakman EC, Stewart DM, Loegering WQ. Identification of physiologic races of *Puccinia graminis* var. *tritici*. USDA ARS Bull. 1962:E617.

[46] Miller JD, Lambert JW. Variability and inheritance of reaction of barley to race 15B of stem rust 1. Agron J. 1955;47:373–7. 10.2134/agronj1955.00021962004700080007x

[47] Steffenson BJ, Case AJ, Pretorius ZA, Coetzee V, Kloppers FJ, Zhou H, et al. Vulnerability of barley to African pathotypes of *Puccinia graminis* f. sp. *tritici* and sources of resistance. Phytopathology. 2017;107:950–62. 28398875 10.1094/PHYTO-11-16-0400-R

[48] Zhou H, Steffenson BJ, Muehlbauer G, Wanyera R, Njau P, Ndeda S. Association mapping of stem rust race TTKSK resistance in US barley breeding germplasm. Theor Appl Genet. 2014;127:1293–304. 24710821 10.1007/s00122-014-2297-8 PMC4035542

[49] Chen S, Zhou Y, Chen Y, Gu J. fastp: an ultra-fast all-in-one FASTQ preprocessor. Bioinformatics. 2018;34:i884–90. 30423086 10.1093/bioinformatics/bty560 PMC6129281

[50] Duplessis S, Cuomo CA, Lin YC, Aerts A, Tisserant E, Veneault-Fourrey C, et al. Obligate biotrophy features unraveled by the genomic analysis of rust fungi. Proc Natl Acad Sci. 2011;108:9166–71. 21536894 10.1073/pnas.1019315108 PMC3107277

[51] Li H, Durbin R. Fast and accurate short read alignment with Burrows-Wheeler transform. Bioinformatics. 2009;25:1754–60. 19451168 10.1093/bioinformatics/btp324 PMC2705234

[52] Dobin A, Davis CA, Schlesinger F, Drenkow J, Zaleski C, Jha S, et al. STAR: ultrafast universal RNA-seq aligner. Bioinformatics. 2013;29:15–21. 23104886 10.1093/bioinformatics/bts635 PMC3530905

[53] Cingolani P, Platts A, Wang LL, Coon M, Nguyen T, Wang L, et al. A program for annotating and predicting the effects of single nucleotide polymorphisms, SnpEff: SNPs in the genome of *Drosophila melanogaster* strain w1118; iso-2; iso-3. Fly. 2012;6:80–92. 22728672 10.4161/fly.19695 PMC3679285

[54] Danecek P, Bonfield JK, Liddle J, Marshall J, Ohan V, Pollard MO, et al. Twelve years of SAMtools and BCFtools. Gigascience. 2021;10:giab008. 33590861 10.1093/gigascience/giab008 PMC7931819

[55] Zheng X, Levine D, Shen J, Gogarten SM, Laurie C, Weir BS. A high-performance computing toolset for relatedness and principal component analysis of SNP data. Bioinformatics. 2012;28:3326–8. 23060615 10.1093/bioinformatics/bts606 PMC3519454

[56] Xavier A, Xu S, Muir WM, Rainey KM. NAM: association studies in multiple populations. Bioinformatics. 2015;31:3862–4. 26243017 10.1093/bioinformatics/btv448

[57] Galili T. Dendextend: an R package for visualizing, adjusting and comparing trees of hierarchical clustering. Bioinformatics. 2015;31:3718–20. 26209431 10.1093/bioinformatics/btv428 PMC4817050

[58] Gu Z, Gu L, Eils R, Schlesner M, Brors B. Circlize implements and enhances circular visualization in R. Bioinformatics. 2014;30:2811–2. 24930139 10.1093/bioinformatics/btu393

[59] Bradbury PJ, Zhang Z, Kroon DE, Casstevens TM, Ramdoss Y, Buckler ES. TASSEL: software for association mapping of complex traits in diverse samples. Bioinformatics. 2007;23:2633–5. 17586829 10.1093/bioinformatics/btm308

[60] Wang J, Zhang Z. GAPIT Version 3: boosting power and accuracy for genomic association and prediction. Genomics Proteomics Bioinformatics. 2021;19:629–40. 34492338 10.1016/j.gpb.2021.08.005 PMC9121400

[61] Zhang Z, Ersoz E, Lai CQ, Todhunter RJ, Tiwari HK, Gore MA, et al. Mixed linear model approach adapted for genome-wide association studies. Nat Genet. 2010;42:355–60. 20208535 10.1038/ng.546 PMC2931336

[62] Huang M, Liu X, Zhou Y, Summers RM, Zhang Z. BLINK: a package for the next level of genome-wide association studies with both individuals and markers in the millions. Gigascience. 2019;8:giy154. 30535326 10.1093/gigascience/giy154 PMC6365300

[63] Catanzariti AM, Dodds PN, Lawrence GJ, Ayliffe MA, Ellis JG. Haustorially expressed secreted proteins from flax rust are highly enriched for avirulence elicitors. Plant Cell. 2006;18:243–56. 16326930 10.1105/tpc.105.035980 PMC1323496

[64] Liu C, Pedersen C, Schultz-Larsen T, Aguilar GB, Madriz-Ordeñana K, Hovmøller MS, et al. The stripe rust fungal effector PEC 6 suppresses pattern-triggered immunity in a host species-independent manner and interacts with adenosine kinases. New Phytol. 2016. 10.1111/nph.14034. 27252028 10.1111/nph.14034

[65] Tian Y, Zhan G, Chen X, Tungruentragoon A, Lu X, Zhao J, et al. Virulence and simple sequence repeat marker segregation in a *Puccinia striiformis* f. sp. *tritici* population produced by selfing a Chinese isolate on Berberis shensiana. Phytopathology. 2016;106:185–91. 26551448 10.1094/PHYTO-07-15-0162-R

[66] Yuan C, Wang M, Skinner DZ, See DR, Xia C, Guo X, Chen X. Inheritance of virulence, construction of a linkage map, and mapping dominant virulence genes in *Puccinia striiformis* f. sp. *tritici* through characterization of a sexual population with genotyping-by sequencing. Phytopathology. 2018;108:133–41. 28876207 10.1094/PHYTO-04-17-0139-R

[67] Xia C, Lei Y, Wang M, Chen W, Chen X. An avirulence gene cluster in the wheat stripe rust pathogen (*Puccinia striiformis* f. sp. *tritici*) identified through genetic mapping and whole-genome sequencing of a sexual population. mSphere. 2020;5:e00128-20. 32554716 10.1128/mSphere.00128-20 PMC7300351

[68] Wang MN, Chen XM. Barberry does not function as an alternate host for *Puccinia striiformis* f. sp. *tritici* in the US Pacific Northwest due to teliospore degradation and barberry phenology. Plant Dis. 2015;99:1500–6. 30695954 10.1094/PDIS-12-14-1280-RE

[69] Korte A, Farlow A. The advantages and limitations of trait analysis with GWAS: a review. Plant Methods. 2013;9:1–9. 23876160 10.1186/1746-4811-9-29 PMC3750305

[70] Ball RD. Designing a GWAS: power, sample size, and data structure. In: Gondro C, Van der Werf J, Hayes B, editors. Genome-wide association studies and genomic prediction. New Jersey: Humana press; 2013. p. 37–98. 10.1007/978-1-62703-447-0_3 23756887

[71] Xia C, Wang M, Cornejo OE, Jiwan DA, See DR, Chen X. Secretome characterization and correlation analysis reveal putative pathogenicity mechanisms and identify candidate avirulence genes in the wheat stripe rust fungus *Puccinia striiformis* f. sp. *tritici*. Front Microbiol. 2017;8:2394. 29312156 10.3389/fmicb.2017.02394 PMC5732408

[72] Li Y, Xia C, Wang M, Yin C, Chen X. Whole-genome sequencing of *Puccinia striiformis* f. sp. *tritici* mutant isolates identifies avirulence gene candidates. BMC Genomics. 2020;2020(21):1–22. 10.1186/s12864-020-6677-y PMC7085141 32197579

[73] Plissonneau C, Benevenuto J, Mohd-Assaad N, Fouché S, Hartmann FE, Croll D. Using population and comparative genomics to understand the genetic basis of effector driven fungal pathogen evolution. Front Plant Sci. 2017;8:119. 28217138 10.3389/fpls.2017.00119 PMC5289978

[74] Rouse MN, Stoxen S, Chen X, Szabo LJ, Jin Y. Diverse stem rust races found in a single field in Washington, USA. Phytopathology. 2009;99:S111.

[75] Bhatia D, Wing RA, Singh K. Genotyping by sequencing, its implications and benefits. Crop Improv. 2013;40:101–11.

[76] Biesecker LG, Shianna KV, Mullikin JC. Exome sequencing: the expert view. Genome Biol. 2011;12:1–3. 10.1186/gb-2011-12-9-128 PMC3308041 21920051

[77] Everhart S, Gambhir N, Stam R. Population genomics of filamentous plant pathogens—a brief overview of research questions, approaches, and pitfalls. Phytopathology. 2021;111:12–22. 33337245 10.1094/PHYTO-11-20-0527-FI

[78] Birky CW. Heterozygosity, heteromorphy, and phylogenetic trees in asexual eukaryotes. Genetics. 1996;144:427–37. 8878706 10.1093/genetics/144.1.427 PMC1207515

[79] Bengtsson BO. Genetic variation in organisms with sexual and asexual reproduction. J Evol Biol. 2003;16:189–99. 14635857 10.1046/j.1420-9101.2003.00523.x

[80] de Meeûs T, Balloux F. Clonal reproduction and linkage disequilibrium in diploids: a simulation study. Infect Genet Evol. 2004;4:345–51. 15374532 10.1016/j.meegid.2004.05.002

[81] Tam V, Patel N, Turcotte M, Bossé Y, Paré G, Meyre D. Benefits and limitations of genome-wide association studies. Nat Rev Genet. 2019;20:467–84. 31068683 10.1038/s41576-019-0127-1

[82] Flor HH. The complementary genic systems in flax and flax rust. Adv Genet. 1956;8:29–54. 10.1016/S0065-2660(08)60498-8

[83] Flor HH. Current status of the gene-for-gene concept. Annu Rev Phytopathol. 1971;9:275–96. 10.1146/annurev.py.09.090171.001423

[84] Zhan J, Thrall PH, Papaïx J, Xie L, Burdon JJ. Playing on a pathogen’s weakness: using evolution to guide sustainable plant disease control strategies. Annu Rev Phytopathol. 2015;53:19–43. 25938275 10.1146/annurev-phyto-080614-120040

[85] Schwessinger B, Chen YJ, Tien R, Vogt JK, Sperschneider J, Nagar R, et al. Distinct life histories impact dikaryotic genome evolution in the rust fungus *Puccinia striiformis* causing stripe rust in wheat. Genome Biol Evol. 2020;12:597–617. 32271913 10.1093/gbe/evaa071 PMC7250506

[86] de Jonge R, Peter van Esse H, Maruthachalam K, Bolton MD, Santhanam P, Saber MK, et al. Tomato immune receptor Ve1 recognizes effector of multiple fungal pathogens uncovered by genome and RNA sequencing. Proc Natl Acad Sci. 2012;109:5110–5. 22416119 10.1073/pnas.1119623109 PMC3323992

[87] Hogenhout SA, Van der Hoorn RA, Terauchi R, Kamoun S. Emerging concepts in effector biology of plant-associated organisms. Mol Plant Microbe Interact. 2009;22:115–22. 19132864 10.1094/MPMI-22-2-0115

[88] Lo Presti L, Lanver D, Schweizer G, Tanaka S, Liang L, Tollot M, et al. Fungal effectors and plant susceptibility. Annu Rev Plant Biol. 2015;66:513–45. 25923844 10.1146/annurev-arplant-043014-114623

[89] Dodds PN, Rathjen JP. Plant immunity: towards an integrated view of plant–pathogen interactions. Nat Rev Genet. 2010;11:539–48. 20585331 10.1038/nrg2812

[90] Jones JD, Dangl JL. The plant immune system. Nature. 2006;444:323–9. 17108957 10.1038/nature05286

