# Supplementary figures and images for: Association mapping with a diverse population of Puccinia graminis f. sp. tritici identified avirulence loci interacting with the barley Rpg1 stem rust resistance gene
Source: BMC Genomics. 2024 Aug 1;25:751. doi: 10.1186/s12864-024-10670-y (PMC11295639; doi:10.1186/s12864-024-10670-y)

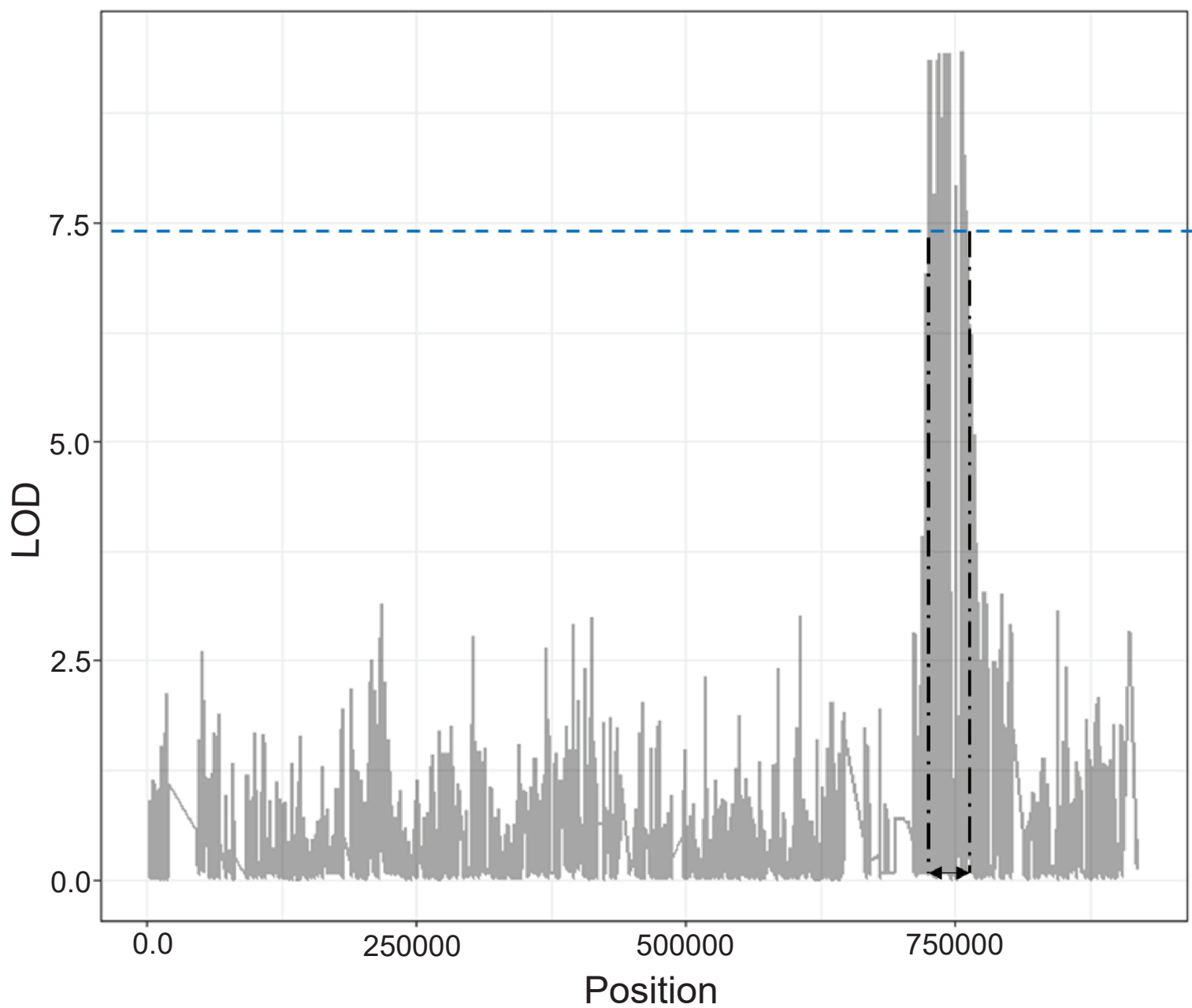

Supplement: Supplementary file 5 — Supplementary Material 5: Supplementary Fig. S1. AvrRpg1A locus identified with Golden promise transgenic line (H228.2c) delimited to 35 kb interval on supercontig2.30. X-axis represents physical position on supercontig2.30. Y-axis represents LOD scores of SNP markers. Blue dotted line represents the significance threshold (LOD = 7.3). Black dotted lines indicate 35 kb interval corresponding to significant markers. [file 12864_2024_10670_MOESM5_ESM.pdf]

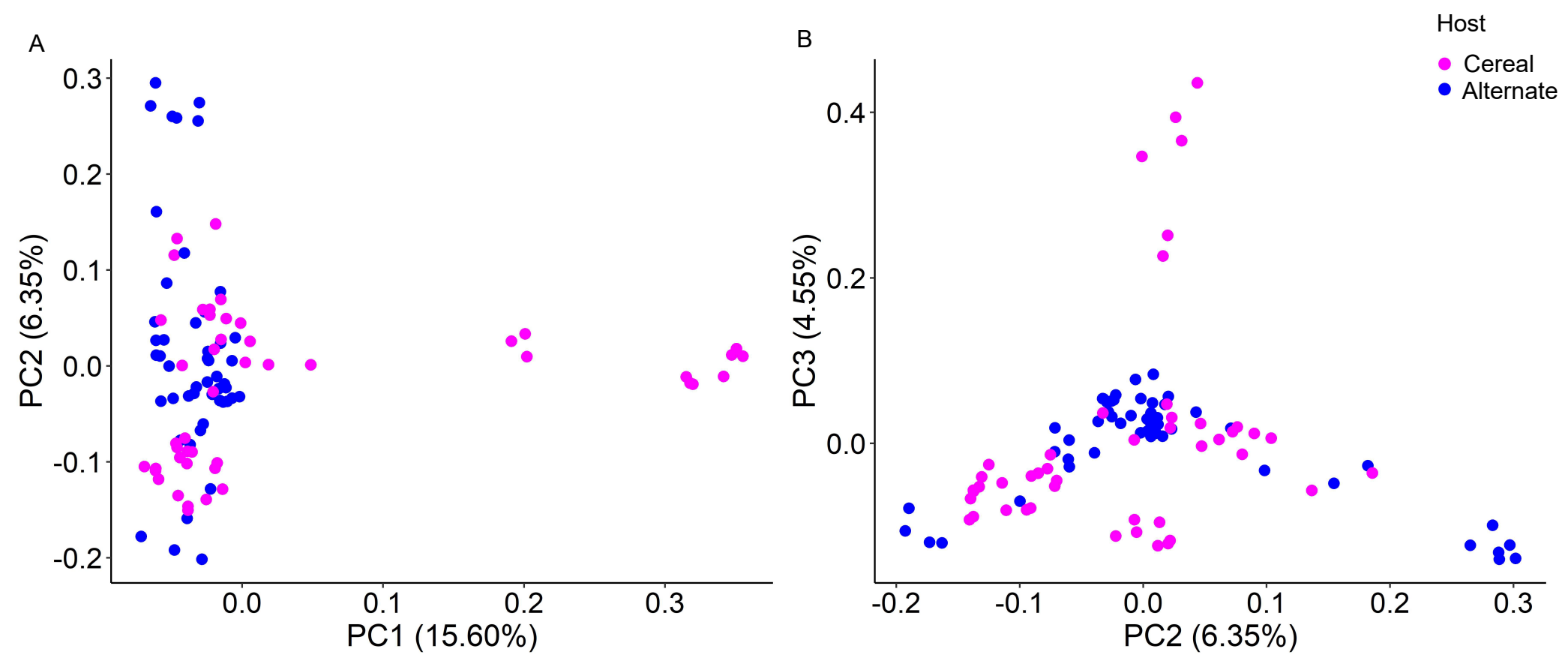

Supplement: Supplementary file 6 — Supplementary Material 6: Supplementary Fig. S2. PCA plots depicting the structure of Pgt population (n = 96). A] PC1 plotted against PC2 B] PC2 plotted against PC3. The percentage of variance explained by PC1, PC2, and PC3 was 15.60%, 6.35%, and 4.55%, respectively. Pink and blue dots indicate isolates collected from cereal and alternate hosts, respectively. [file 12864_2024_10670_MOESM6_ESM.pdf]
